# Chromatin landscape dynamics during reprogramming towards human naïve and primed pluripotency reveals the divergent function of *PRDM1* isoforms

**DOI:** 10.1038/s41420-024-02230-w

**Published:** 2024-11-19

**Authors:** Jianfeng Zhou, Mingyue Guo, Guang Yang, Xinyu Cui, Jindian Hu, Tan Lin, Hong Wang, Shaorong Gao, Cizhong Jiang, Liping Wang, Yixuan Wang

**Affiliations:** 1 Shanghai Key Laboratory of Maternal and Fetal Medicine, Clinical and Translational Research Center of Shanghai First Maternity and Infant Hospital, School of Life Sciences and Technology, Tongji University, 200092 Shanghai, China; 2 Frontier Science Center for Stem Cell Research, Tongji University, 200092 Shanghai, China; 3 The Fifth Affiliated Hospital of Guangzhou Medical University, 510700 Guangzhou, China; 4 Key Laboratory of Spine and Spinal Cord Injury Repair and Regeneration of the Ministry of Education, Orthopaedic Department of Tongji Hospital, Tongji University, 200065 Shanghai, China; 5 Shanghai Tenth People’s Hospital, School of Life Sciences and Technology, Tongji University, 200072 Shanghai, China

**Keywords:** Reprogramming, Pluripotency, Embryonic stem cells, Induced pluripotent stem cells

## Abstract

Induced pluripotent stem cells (iPSCs) technology holds great potential in both scientific research and clinical applications. It enables the generation of naïve and primed iPSCs from various cell types through different strategies. Despite extensive characterizations of transcriptional and epigenetic factors, the intricacies of chromatin landscape dynamics during naïve and primed reprogramming, particularly in humans, remain poorly understood. In this study, we employed ATAC-seq and RNA-seq analyses to delineate and compare the chromatin landscape of naïve and primed pluripotency through the human secondary reprogramming system. Our investigations revealed several key transcriptional and epigenetic factors pivotal for reprogramming-associated chromatin remodeling. Notably, we found two isoforms of *PRDM1*, *PRDM1α,* and *PRDM1β*, bind to distinct genomic loci and play different roles in the naïve reprogramming process. We proposed an auto-regulatory model explaining the distinct functions of *PRDM1α* and *PRDM1β*. Overall, our findings highlight the complexity and diversity of transcription factors in shaping chromatin landscape dynamics and directing the fates of pluripotent cells.

## Introduction

The past decade has witnessed the rapid development of the regenerative medicine field by the advent of induced pluripotent stem cells (iPSCs). These cells offer great promise for basic research and clinical applications [1]. Notably, iPSCs present a solution to the ethical quandaries and immune rejection associated with using human embryonic stem cells (hESCs) derived from human embryos. Several research groups have successfully generated iPSCs from diverse cell types by the ectopic expression of transcription factors or by the chemical stimulation of small molecules [1, 2]. Moreover, two different pluripotent states, naïve and primed, can be captured in vitro, corresponding to the pre- and post-implantation stages of in vivo development, respectively, with naïve pluripotency being considered to possess better developmental potential [3]. Similarly, we can obtain naïve iPSCs and primed iPSCs through somatic cell reprogramming. Despite these advances, challenges persist due to the low efficiency and significant heterogeneity of the reprogramming process, which continues to obscure the underlying mechanisms. Numerous investigations have dissected the cellular changes of the reprogramming both in mice and humans, mostly at transcriptional and epigenetic levels [4–7]. However, the dynamics of the chromatin landscape during naïve and primed iPSC reprogramming is poorly understood, especially in humans. In recent years, some newly developed technologies have made it possible to capture chromatin accessibility across the whole genome, which helps us to understand the intermediate state further and fill the gap in the knowledge of reprogramming mechanisms [8].

In this study, we adopted the high-throughput assay for transposase-accessible chromatin (ATAC-seq) coupled with RNA-sequencing (RNA-seq) to profile the chromatin accessibility landscape and transcriptomic dynamics in our previously established secondary (2°) human reprogramming systems toward naïve and primed pluripotency, respectively [9]. Our analysis identified several pivotal transcription factors and epigenetic regulators that affect reprogramming efficiency, laying the groundwork for further precise manipulations. Importantly, we found the divergent roles of *PRDM1* (positive regulatory domain-containing protein 1) gene isoforms, *PRDM1α* and *PRDM1β*, during the reprogramming towards naïve pluripotency but not primed state. By utilizing CUT&Tag, we discovered that *PRDM1α* and *PRDM1β* target distinct genomic sites, exerting different effects on the target genes. This suggests the possibility of a regulatory yin-yang model between *PRDM1α* and *PRDM1β*, mediated through their interactions with *SPRED2* and *DDAH1*, respectively. In addition, our findings revealed that neither *PRDM1α* nor *PRDM1β* is indispensable to maintaining naïve pluripotency. Taken together, our study demonstrates the crucial role of *PRDM1* in the naïve pluripotency establishment, providing an example to direct the fates of pluripotent cells. The precise regulation of these key transcription factors helps to improve the reprogramming efficiency, obtain higher quality iPSCs, and lay the foundation for clinical application.

## Results

### Chromatin dynamics during human iPSCs reprogramming

**Fig. 1:**
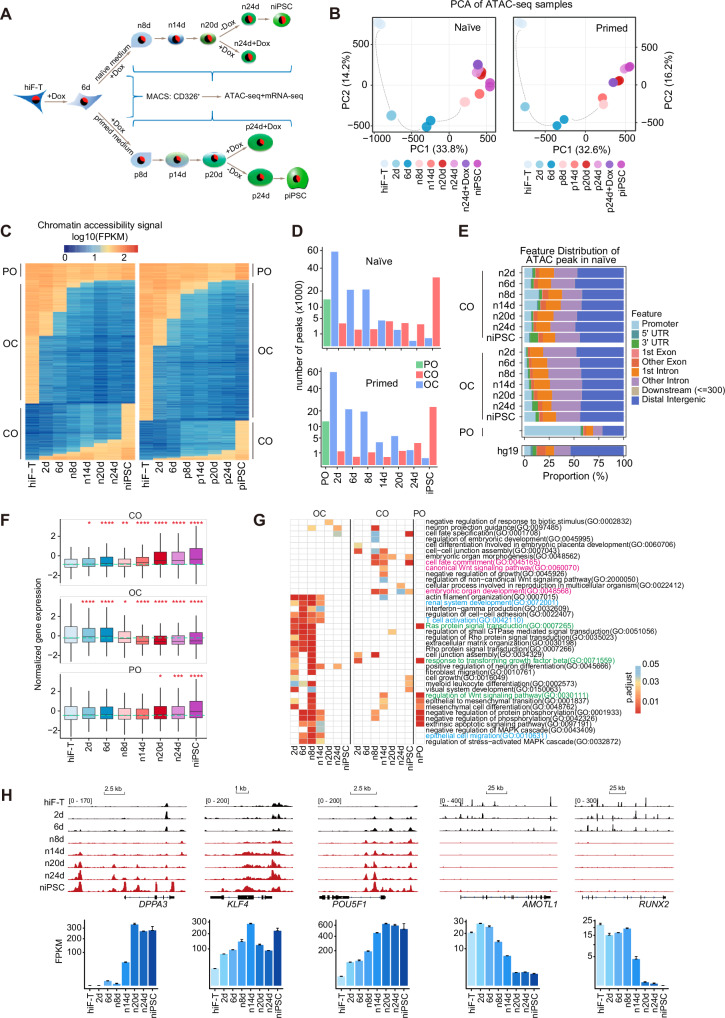
ATAC-seq and mRNA-seq revealed highly dynamic chromatin changes during human naïve and primed reprogramming. **A** Schematic experimental design of human 2° hiF-T (immortalized fibroblasts with inducible *OCT4, SOX2, NANOG, C-MYC* cassette) reprogramming; Fan diagram within nuclei represents proportions of pluripotency states; MACS: magnetic-activated cell sorting. **B** Principal component analysis (PCA) on the ATAC-seq data collected from cells at different stages of naïve and primed reprogramming. **C** Open chromatin regions were categorized into three types: consistently accessible throughout reprogramming as permanently open (PO), regions transitioning from closed in fibroblasts to open as the reprogramming proceeded as closed to open (CO), and the converse as open to closed (OC). Log10-transformed FPKM values are used to represent the degree of chromatin accessibility at a given region. **D** Numbers of PO, CO, and OC regions at each stage of naïve and primed reprogramming. **E** Enrichment of naïve reprogramming ATAC-seq peaks at different genomic features. **F** Box plots depicting the normalized expression levels of genes within 10 kb of PO, CO, and OC regions in naïve reprogramming. The green dashed line indicates the median gene expression level corresponding to the hiF-T stage. Mann–Whitney *U* test versus the levels in hiF-T, *p < 0.05, **p < 0.01, ***p < 0.001, ****p < 0.0001. **G** Gene ontology (GO) analysis on all genes within 10 kb of the PO, CO, and OC regions in naïve reprogramming. **H** Representative gene expression and chromatin accessibility in naïve reprogramming; pluripotency genes, *DPPA3*, *KLF4*, *POU5F1*; somatic genes, *AMOTL1*, *RUNX2*.

To investigate the chromatin dynamics during somatic cell reprogramming toward different states of pluripotency, we employed the 2° human induced pluripotent stem cell (hiPSC) reprogramming system that we previously developed [9]. This system involves the transfection of somatic cells with gene cassettes containing doxycycline (Dox)-inducible Yamanaka factors, facilitating the generation of clonal iPSCs. These iPSCs can subsequently be differentiated back into somatic cells, which possess the potential for a second reprogramming upon Dox reinduction. By introducing constitutive expression of human telomerase reverse transcriptase (TERT) into our 2°-inducible fibroblasts system (2° hiF-T), we successfully overcame the challenges of inefficiency and heterogeneity encountered in the primary reprogramming system. We directed these 2° hiF-T cells towards naïve and primed states of pluripotency (Fig. S1A). We harvested CD326^+^ cells, the putative pluripotent intermediates, at days 6, 8, 14, 20, and 24, alongside the initial fibroblasts (hiF-T) and the final iPSCs, which were subjected to ATAC-seq and RNA-seq analyses (Fig. 1A and Supplementary Table 1).

Additionally, to interrogate the effects of sustained exogenous Yamanaka factor expressions post-day 20, we collected samples with continued Dox supplement until day 24 (Fig. 1A). ATAC-seq signals revealed a strong correlation among day 24 replicates (Fig. S1B). Moreover, samples with or without Dox from day 20 to day 24 demonstrated substantial correlation (Fig. S1B), indicating that by 20 days of reprogramming, the emerging iPSCs attained a relatively stable chromatin state, irrespective of continuous Yamanaka factor expression.

Chromatin accessibility dynamics appeared congruent in both naïve and primed reprogramming paths, with day 6 marking a significant juncture coinciding with medium changes (Fig. 1B). Despite this similarity, transcriptomic landscapes diverged significantly. A pronounced shift in transcriptome profiles was noted until day 14 for naïve reprogramming, whereas the corresponding change for primed reprogramming occurred around day 8 (Fig. S1C). By day 20 and day 24, the transcriptomic signatures of reprogrammed cells displayed minimal divergence from those of stable, cultured iPSCs (Fig. S1C). There are some differences between naïve 20/24 and naïve iPSCs (Fig. S1C), which we believe are primarily due to poor genomic stability during prolonged 5iLAF naïve medium culture [10]. Using ATAC-seq data, peaks in open chromatin regions were identified (Fig. S1D). The temporal tracking of the same genomic loci revealed highly dynamic chromatin accessibility throughout both naïve and primed reprogramming trajectories (Fig. S2A). We categorized regions consistently accessible throughout reprogramming as permanently open (PO), regions transitioning from closed in fibroblasts to open as the reprogramming proceeded as closed to open (CO), and the converse as open to closed (OC) (Fig. 1C). Regarding the abundance of ATAC-seq peaks, the OC regions consistently outnumbered the CO regions until day 20 during naïve reprogramming, a trend echoed in the primed reprogramming (Fig. 1D). Also, the number of CO regions progressively increased over time during both the naïve and primed stages, reaching its peak at the iPSC stage (Fig. 1D). Genomic feature annotations exhibited a comparable distribution of peaks in all clusters (Fig. 1E). In addition, we also observed a positive correlation between chromatin accessibility and gene expression levels (Fig. S1E) as previously reported [11], reinforcing the interplay between the epigenetic landscape and transcriptional activity during reprogramming.

We further annotated the defined PO, CO, and OC regions to the nearest genes within a 10-kilobase (kb) distance and quantified the associated gene expression levels (Fig. 1F and S2B). During naïve reprogramming, we observed a consistent and significant upregulation of gene expression in the CO regions at each examined time point compared to the initial hiF-T cells. In contrast, genes associated with the OC regions demonstrated a marked decrease in expression starting from day 8, while genes in the PO regions exhibited negligible changes in expression levels (Fig. 1F and S2C). During primed reprogramming, the gene expression patterns in the CO regions mirrored those observed during naïve reprogramming (Fig. S2B). However, gene expression within the OC regions was slightly up-regulated, while PO region-associated gene expression was significantly elevated (Fig. S2B).

Gene Ontology (GO) analysis for the genes in ATAC peaks revealed distinct enrichment (Fig. 1G and S2D): In the CO regions, the associated genes are primarily involved in pluripotency or early embryonic development processes. These genes drive successful reprogramming, as evidenced by GO terms such as “cell fate commitment”, “regulation of stem cell proliferation”, and “regulation of embryonic development”, which are well-aligned with established knowledge. Conversely, in the OC regions, the corresponding genes are associated with somatic cell lineages and biological activities that reflect a differentiated state. GO terms enriched in these regions, such as “positive regulation of neuron differentiation”, “T cell activation”, and “fibroblast migration” (Fig. 1G and S2D), further support this differentiated signature. For the PO regions, the enriched terms, including “response to transforming growth factor beta”, “extrinsic apoptotic signaling pathway”, and “cell growth”, align with the involvement of these genes in fundamental biological activities and key signaling pathways. Specifically, pluripotency-specific genes such as *DPPA3*, *KLF4*, and *POU5F1*, along with developmental patterning genes like *LEFTY1* and *FOXA2*, gradually acquired an open chromatin state throughout reprogramming. Conversely, the chromatin surrounding those somatic genes, like *AMOTL1* and *RUNX2*, progressively condensed, indicating a gradual shutdown of their transcriptional activity (Fig. 1H and S2E). We also validated these changes on the protein level (Fig. S2F, G). These results fully demonstrate the complexity and diversity of the human somatic reprogramming process.

### Distinct trajectories between naïve and primed hiPSCs reprogramming

**Fig. 2:**
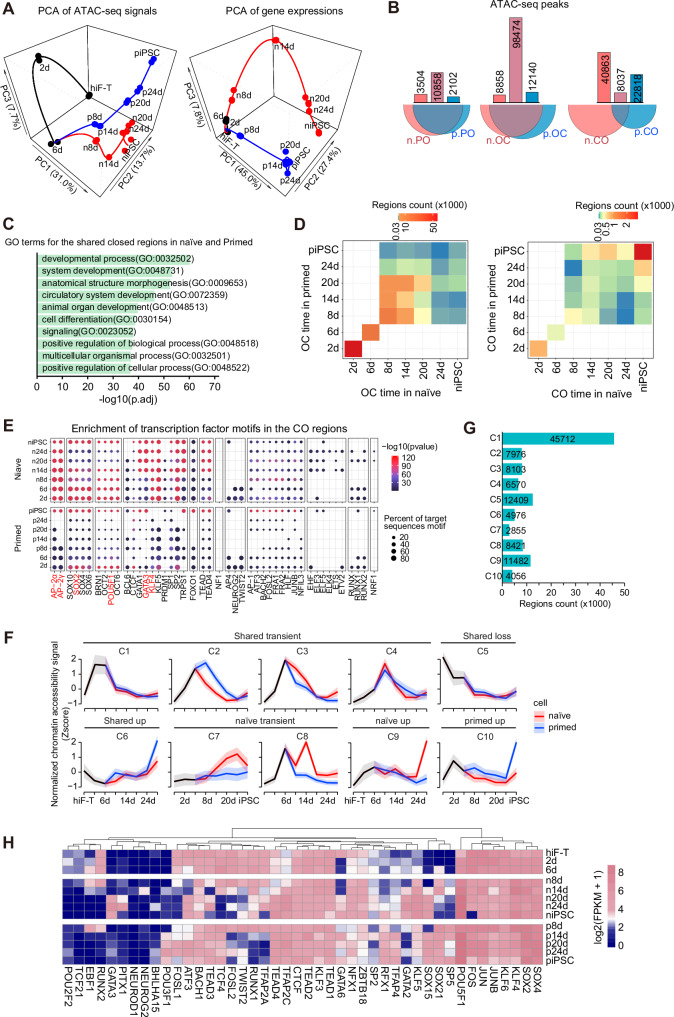
Chromatin accessibility, transcriptome, and TF motif analysis revealed distinct trajectories between naïve and primed hiPSCs reprogramming. **A** Three-dimensional PCA of the integrated ATAC-seq (left) and RNA-seq (right) datasets from cells at different stages of naïve and primed reprogramming. **B** Venn diagram showing the overlap between naïve and primed reprogramming in PO, OC, and CO regions from all time points. **C** GO analysis on all genes within the shared closed regions of naïve and primed reprogramming. **D** Heatmap illustrating the corresponding temporal relationship of chromatin changes in the shared OC regions (left) and CO regions (right) between naïve and primed reprogramming, derived from the overlapping peaks in (**B**). Color intensity indicates the number of peaks simultaneously open and closed at corresponding time point in naïve and primed conditions. **E** The bubble chart depicting the enrichment of transcription factor motifs at the CO regions in naïve and primed reprogramming. **F** Unsupervised clustering analysis of transiently opened or closed chromatin regions shown in Fig S2A. These clusters are divided into 6 categories: shared transient (C1–C4), shared loss (C5), shared up (C6), naïve transient (C7, C8), naïve up (C9), and primed up (C10). Solid lines and ribbons represent the mean of standardized ATAC-seq signals across clusters ± s.d. **G** The bar plot showing the count of each cluster given in Fig. 2F. **H** Heatmap displaying the gene expression levels corresponding to the most significantly enriched TF motifs in each cluster as depicted in (**F**).

To concisely elucidate the divergent paths of naïve and primed reprogramming, RNA-seq and ATAC-seq data from both reprogramming strategies were integrated using a three-dimensional principal component analysis (PCA) (Fig. 2A). Analysis revealed distinct trajectories, underscoring substantial changes that affirm the disparate nature of these two reprogramming systems (Fig. 2A). Notably, chromatin accessibility began to differ on day 8, post-medium alteration, which preceded the dramatic transcriptome discrepancies that emerged around day 14 (Fig. 2A). This temporal shift indicates that alterations in chromatin accessibility are an early indicator of the ensuing transcriptional changes. The positive correlation between ATAC-seq signals and gene expression levels (Fig. S1E, S3A) further indicated the functional significance of the chromatin accessibility dynamics that we observed. Intriguingly, our findings suggested that a primed state is not a prerequisite for achieving naïve pluripotency, corresponding to an earlier developmental stage than the primed state.

We also compared the similarities and differences between the naïve and primed reprogramming processes in open chromatin regions from different perspectives. The PO regions, as well as OC regions in naïve reprogramming, overlapped with more than two-thirds of the primed PO regions, indicating a shared chromatin landscape (Fig. 2B). However, the CO regions in the naïve reprogramming system exhibited minimal overlap with the primed CO regions (~11%), mirroring the distinctive cellular identities achieved by each reprogramming approach (Fig. 2B). The number of genes within these regions also displayed a similar trend (Fig. S3B). Under the force of the OSKM reprogramming factors, chromatins associated with somatic identity were rapidly turned off in both naïve and primed reprogramming (Fig. 2D). In contrast, in the common CO regions, naïve and primed reprogramming did not reveal relatively more co-opened pluripotent regions until the iPSC stage (Fig. 2D), which is consistent with the number of open chromatin regions at different stages shown in Fig. 1D. This pattern indicated that gene interactions and regulatory mechanisms within CO regions were pivotal in dictating the reprogramming processes.

Next, we performed transcription factors (TFs) motif enrichment analysis within the identified chromatin dynamic regions. We observed several types of TFs enriched in the CO or OC regions at different time points during naïve and primed reprogramming, with apparent discrepant TF family preference between CO and OC regions (Fig. 2E and S3C). TFs like *TWIST2*, *AP-1*, *FOSL2*, *JUNB*, and *RUNX* were mainly enriched in the OC regions. Notably, *NRF1* demonstrated a unique enrichment pattern, specifically abundant in the PO regions (Fig. S3C). Moreover, in the OC and PO regions, the enrichment scores of each TF showed no significant difference in naïve reprogramming or primed reprogramming (Fig. S3C). As for the CO regions, it is important to note that the degree of enrichment significance for some TF families, including *AP-2*, *SOX2*, *POU5F1*, *GATA3*, and *KLF4*, varies between naïve and primed reprogramming, suggesting their differential regulatory impacts on the reprogramming processes. (Fig. 2E).

In our analysis, we classified genomic regions into three categories (CO, OC, PO) based on their persistent accessibility status (Fig. 1C), indicating that once altered, these regions remained consistently open or closed until the end of reprogramming. This classification was referred to as the pattern style. Contrarily, we observed high chromatin accessibility dynamics, with numerous regions transiently altering status (Fig. S2A) (termed non-pattern style henceforth). Through unsupervised clustering of these non-pattern regions, we identified 10 clusters reflecting chromatin accessibility trends, which were further categorized into 6 groups: shared transient (C1-C4), shared loss (C5), shared up (C6), naïve transient (C7, C8), naïve up (C9), and primed up (C10) (Fig. 2F, G). Correspondingly, the trends in gene expression adjacent to these non-pattern regions followed a similar trend to the dynamics of chromatin accessibility (Fig. 2F and S3D).

Motif analysis was also performed across these non-pattern regions. Clusters C1–C4 (shared transient) were significantly enriched for both pluripotent TFs, such as *POU5F1* and *SOX2*, and somatic TFs, including *TCF21* and *TWIST2* (Fig. S3E). This observation was consistent with a previously established model proposing that during the initial phase of reprogramming, somatic TFs were redistributed by core pluripotent TFs from their original somatic enhancer loci to transient opened loci to repress the somatic program [12]. The expression changes of the TFs exhibited a different pattern between naïve and primed reprogramming, suggesting the same TF may function as distinct roles during two reprogramming processes (Fig. 2H). Using somatic reprogramming as an example, these findings highlight the significant differences in developmental and differentiation trajectories between the naïve and primed states of pluripotency.

### Identifying epigenetic factors responsible for human naïve reprogramming

**Fig. 3:**
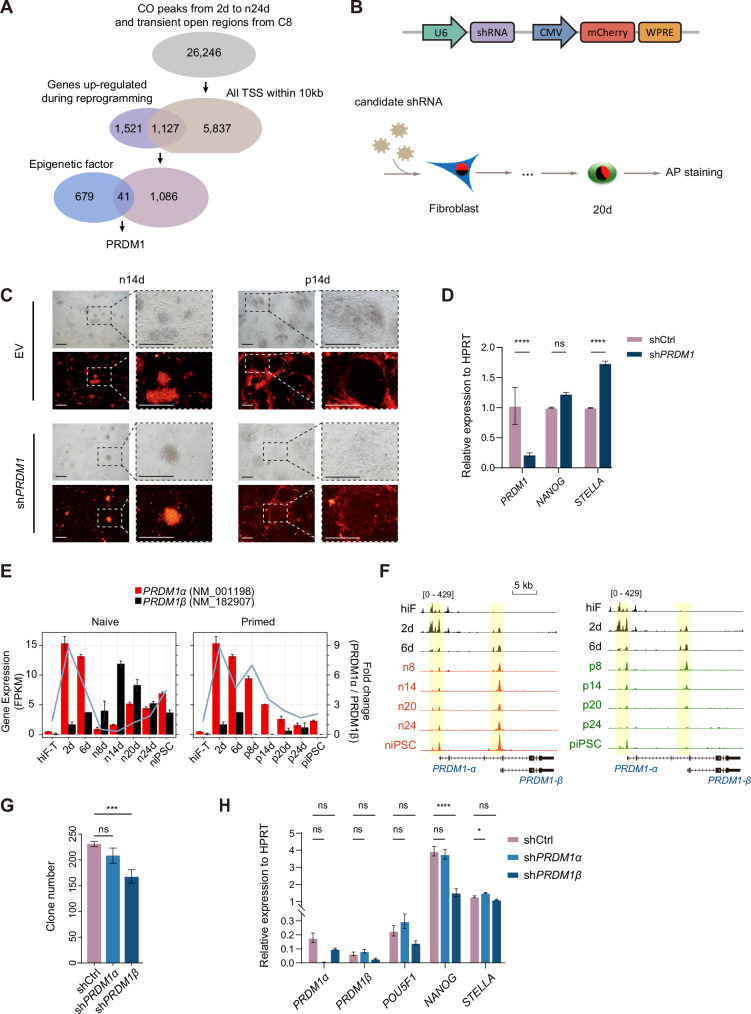
Two *PRDM1* isoforms exhibit distinct effects during naïve reprogramming. **A** Schematic of the strategy used to identify *PRDM1*. The CO and C8 peaks were annotated to 5837 TSSs within 10 kb, of which 1127 genes were upregulated during naïve reprogramming. After the intersection with the epigenetic factors database, 41 candidate genes were selected as potential regulators. **B** Schematic experimental design of knocking down selected candidate genes with short hairpin RNA (shRNA) during reprogramming. **C** Bright field and fluorescent images of reprogramming intermediates in n14d and p14d upon sh*PRDM1*, empty vector was set as Ctrl. 3-5 pictures were taken. The experiment was repeated three times. Scale bar, 100μm. **D** Relative expression of *PRDM1*, *NANOG* and *STELLA* upon sh*PRDM1* via qPCR in naïve reprogramming. *n* = 3; Two-way ANOVA, ****, adjusted *p*-value < 0.0001. **E** Gene expression of *PRDM1α* and *PRDM1β* (bar plot), and the fold difference (blue line) between them during reprogramming. **F** The snapshots of the browser view showing chromatin accessibility dynamics near *PRDM1α* and *PRDM1β* during reprogramming. **G** Clone numbers under sh*PRDM1α* and sh*PRDM1β* treatments in naïve reprogramming. The experiment was repeated three times; one-way ANOVA, ** adjusted *p*-value = 0.0013. **H** Relative expression of *PRDM1α*, *PRDM1β*, *POUF51*, *NANOG*, and *STELLA* under sh*PRDM1α* and sh*PRDM1β* treatments in naïve reprogramming. *n* = 3; Two-way ANOVA, *, adjusted *p*-value = 0.0478; ****, adjusted *p*-value < 0.0001.

Our analysis revealed significant distinctions in the CO regions between naïve and primed reprogramming (Fig. 2B, D). In human reprogramming studies, most of the work focuses on primed states, and less on naïve states, so we choose to focus on the naïve reprogramming process. As the complex network of gene interactions and regulatory mechanisms within these CO regions may play a pivotal role in dictating the success of reprogramming, we then sought to explore the involvement of epigenetic factors in modulating chromatin dynamics during naïve reprogramming. To this end, CO peaks (26246 regions, Supplementary Table 2) across various stages of reprogramming day 2, day 6, day 8, and day 24 from ATAC-seq data, including the transient open peaks identified in C8 (Fig. 2F and S3C), were collected. By focusing on genes with their transcription start site (TSS) located within 10 kb of these 26246 CO regions, we refined our search to those up-regulated genes during naïve reprogramming (Supplementary Table 3). Further refinement using an annotated library of epigenetic factors [13] led us to identify 41 candidate genes likely influencing chromatin accessibility during human naïve reprogramming (Fig. 3A).

These candidate genes were categorized into 4 groups according to their expression patterns during naïve reprogramming (Fig. S4A). Group 1 genes, including *ARRB1*, *RNF2*, *PAXIP1*, *BRWD*3, *PCGF6*, and *PRDM1*, exhibited a progressive up-regulation along with the reprogramming process. Conversely, group 2 genes remained consistently expressed, showing negligible expression changes. Group 3 genes were characterized by a sudden surge in expression at the later stages of reprogramming, with notable members such as *DPPA3*, *DNMT3B*, and *TET1* reported for their critical roles in human naïve pluripotency. Group 4 genes displayed low and stable expression levels. Given these insights, we focused on group 1 genes for further investigation. Moreover, *TFAP2A* and *TFAP2C* were identified to be potential TFs that could regulate reprogramming chromatin remodeling (Fig. 2E), which were also included in our candidate regulators for further analyses. These identified candidate genes provide strong material for further investigating the critical role of key epigenetic factors in reprogramming.

### *TFAP2C* was required for naïve reprogramming

For functional validations, we designed specific short hairpin RNAs (shRNAs) to knock down these candidate genes during naïve programming (Fig. 3B). Primed reprogramming was also performed as another control condition. Compared to the control, knocking down *TFAP2A* or *TFAP2C* affected naïve reprogramming and had no noticeable impact on primed reprogramming (Fig. S4B), which was consistent with previous studies on *TFAP2A* and *TFAP2C* that could regulate pluripotent programs [14–16]. *TFAP2C* has also been reported to be crucial in regulating human naïve pluripotency maintenance [17]. In our analysis, the promoter and enhancer regions of the *TFAP2C* locus gradually acquired chromatin accessibility (Fig. S4C), and the expression level of *TFAP2C* elevated along with the naïve reprogramming process (Fig. S4D). *TFAP2C* knockdown during naïve reprogramming led to fewer iPSC colonies (Fig. S4E). Detailed inspection showed that the knockdown of *TFAP2C* impaired naïve iPSCs but had a negligible impact on primed iPSCs cellular phenotypes (Fig. S4F, upper panel). In contrast, its silencing during naïve reprogramming led to decreased expression of naïve pluripotency-specific genes (Fig. S4G). These results confirmed the significance of *TFAP2C* in establishing naïve pluripotency and further substantiated the efficacy of our selection strategy.

### *PRDM1* exhibits dual characters during naïve reprogramming

Compared to the non-targeting control shRNAs, knocking down *PRDM1* or *PAXIP1* significantly affected naïve reprogramming, with the latter resulting in almost no colony (Fig. S4B). Similar phenotypes were observed in *PAXIP1*-knockdown during primed reprogramming, although they exhibited a modest effect (Fig. S4B). Unlike *PAXIP1* knockdown, we could keep and harvest iPSC colonies upon *PRDM1* silence for further characterization.

Beyond the colony formation capability, the morphology of these colonies provided additional insights. Notably, silencing *PRDM1* in naïve reprogramming significantly reduced the number of iPSC colonies, albeit with improved morphological characteristics (Fig. 3C, D). In contrast, *PRDM1* knockdown during primed reprogramming did not elicit a similar phenotype (Fig. 3C). Known as B lymphocyte-induced maturation protein-1 (*BLIMP-1*), *PRDM1* plays a critical role in the differentiation and maturation of various immune and germline cells [18–21]. It modulates gene expression as a transcriptional regulator by engaging with histone-modifying enzymes, typically leading to transcriptional silencing [22–26]. Despite its well-established roles, the involvement of *PRDM1* in both naïve and primed reprogramming processes remains unexplored.

Intriguingly, a detailed analysis of the *PRDM1* gene locus revealed the presence of two splicing variants, *PRDM1α* and *PRDM1β*, with the latter being driven by an alternative promoter located in the third exon of *PRDM1α* (Fig. S4H). The expression of the two isoforms during somatic reprogramming was inversely correlated, with *PRDM1α* expression gradually decreasing along with primed reprogramming and *PRDM1β* expression being consistently low (Fig. 3E, F), chromatin accessibility within the gene loci displayed a similar trend (Fig. 3F). Conversely, during naïve reprogramming, a reciprocal expression pattern emerged: an increase in *PRDM1α* expression was accompanied by a decrement in *PRDM1β* expression, indicative of an exclusive expression relationship (Fig. 3E, F). As the shRNA sequences designed initially targeted the common exons of both *PRDM1α* and *PRDM1β* transcripts, leading to the simultaneous knockdown of both isoforms, the phenotypes observed in naïve reprogramming with fewer quantity and better quality of colonies could be attributed to the disparate roles of the *PRDM1* isoforms.

To test this hypothesis, shRNA sequences specific to the unique regions of *PRDM1α* and *PRDM1β* were designed and utilized for isoform-specific knockdowns to dissect their contributions during naïve reprogramming. Suppression of *PRDM1α* did not substantially alter the quantity of obtained iPSC colonies relative to the control group (Fig. 3G and S4F). Yet, improvements in colony morphology were evident alongside a modest upregulation of the pluripotent genes *POU5F1* and *STELLA* (Fig. 3H and S4F). Conversely, targeting *PRDM1β* alone resulted in a significant decrease in the quantity of iPSC colonies (Fig. 3G and S4F) and concomitantly reduced the expression of pluripotency markers *NANOG* and *STELLA* (Fig. 3H). These results suggest that a single *PRDM1* gene can have multiple distinct roles in the reprogramming process, broadening the scope of gene function research.

### *PRDM1α* nor *PRDM1β* was indispensable to maintaining naïve pluripotency

**Fig. 4:**
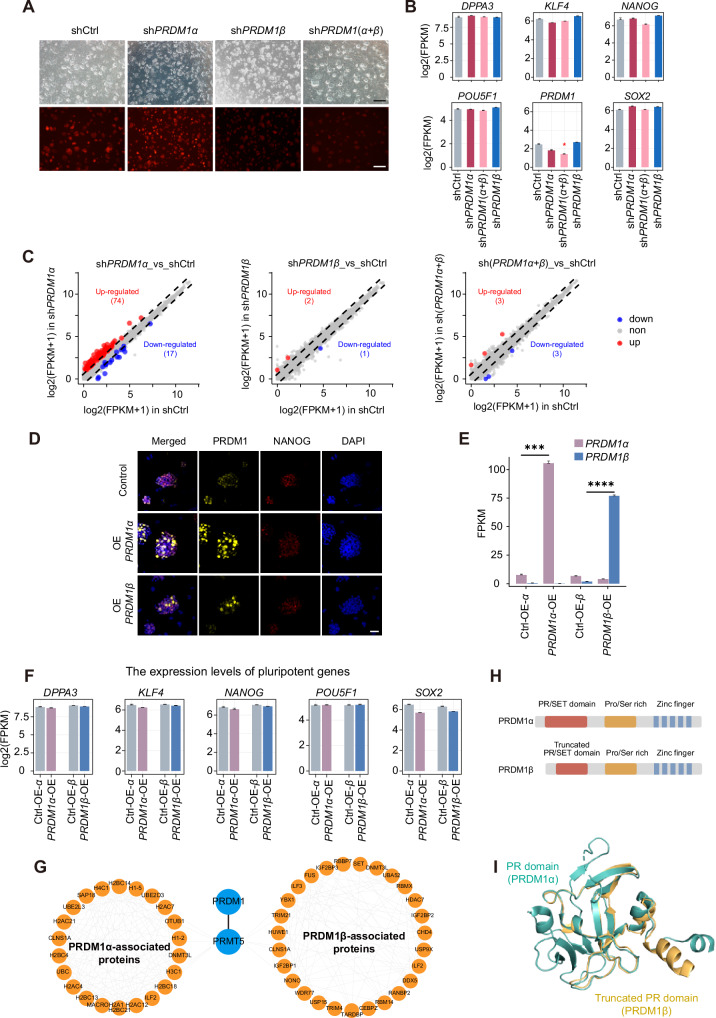
Knockdown or overexpression of *PRDM1α* and *PRDM1β* showed negligible effects on naïve pluripotency maintaining. **A** Bright field and fluorescent images of naïve hESCs in Ctrl (empty vector), sh*PRDM1α*, sh*PRDM1β*, and sh*PRDM1(α* + *β*). Scale bar, 200μm. **B** Expression levels of pluripotent genes upon *PRDM1* knockdown. T-test versus the levels in shCtrl, *p < 0.05, and empty means non-significant. **C** Scatter plot of differential expression genes between sh*PRDM1α*, sh*PRDM1β* and sh*PRDM1(α* + *β*) versus shCtrl. Differential expression genes were defined with fold-change > 2 and *q* value < 0.05 using the R package Ballgown. **D** Immunofluorescence images of Ctrl (empty vector), OE PRDM1α, OE PRDM1β in naïve hESCs. Scale bar, 50μm. **E** Expression levels of *PRDM1α* and *PRDM1β* upon their overexpression. **F** Expression levels of pluripotent genes upon the overexpression of *PRDM1α* or *PRDM1β*. All the genes showed no significant expression change versus the control group using the *T* test. **G** PRDM1α and PRDM1β specific proteome in naïve hESCs. **H** Protein structures of PRDM1α and PRDM1β. **I** PR domain structure prediction of PRDM1β and structure compared with PRDM1α.

Next, we investigated whether the two isoforms of *PRDM1* function in maintaining naïve pluripotency. Knockdown of *PRDM1α* and *PRDM1β* separately or collectively in established naïve human embryonic stem cell (hESC) lines did not result in significant morphological changes (Fig. 4A). RNA-seq analysis revealed a minimal impact on gene expression, with no substantial changes in the expression levels of critical naïve pluripotency-associated genes such as *DPPA3*, *KLF4*, and *NANOG* (Fig. 4B, C).

Meanwhile, efforts to establish naïve hESC lines with inducible overexpression systems for *PRDM1α* and *PRDM1β* faced challenges. Despite utilizing standard single-cell cloning steps, the obtained cell lines showed substantial heterogeneity in protein overexpression levels, especially for *PRDM1β* (Fig. 4D). Achieving *PRDM1β* overexpression proved to be especially difficult, resulting in only a slight increase in protein expression (Fig. S5A, B, Supplementary Original Blots). This suggests the existence of a regulatory mechanism limiting PRDM1β protein overexpression. However, this restriction does not extend to *PRDM1α* and *PRDM1β* mRNAs (Fig. 4E). RNA-seq analysis for these inducible overexpression lines showed minimal differential gene expression (Fig. S5C, D), with no effect on the expression of naïve pluripotency-associated markers such as *DPPA3*, *KLF4* and *NANOG* or shared pluripotency genes *POU5F1* and *SOX2* (Fig. 4F). Moreover, collective comparisons of transcriptomic profiles from knockdown and overexpression lines with published data on naïve hESCs revealed no significant discrepancies (Fig. S5E). Stemness analysis suggested that genes up-regulated following *PRDM1α* knockdown were more associated with stem cell signatures than other groups (Fig. S5F, G).

We also performed immunoprecipitation-mass spectrometry (IP-MS) on the *PRDM1α* and *PRDM1β* overexpression cell lines. After eliminating the IgG background data, we identified 355 and 323 interacting proteins for PRDM1α and PRDM1β, respectively, with 43 proteins shared between them (Fig. S5H and Supplementary Table 4). GO analysis of these interactions revealed that the proteins specifically interacting with PRDM1α and PRDM1β were involved in similar biological processes, notably in translation, gene expression, and ribonucleoprotein complex biogenesis (Fig. S5I, J), underlying their crucial roles in these processes. PRMT5, a known PRDM1 cofactor, featured prominently among the interacting proteins for both PRDM1 isoforms, suggesting that this interaction does not rely on the intact PR domain (Fig. 4G, H). Structural predictions for PRDM1β using AlphaFold2, compared to the PR domain structure of PRDM1α (PRDM1, Protein Data Bank (PDB): 3DAL), revealed the absence of 5 β-sheets in PRDM1β’s PR domain (Fig. 4I), highlighting structural variances that merit further investigation. In conclusion, our findings indicate that neither *PRDM1α* nor *PRDM1β* is essential for maintaining naïve pluripotency.

### *PRDM1α* and *PRDM1β* targeted different genomic loci

To investigate the binding patterns of *PRDM1α* and *PRDM1β* during reprogramming, we employed the recently developed Cleavage Under Targets & Tagmentation (CUT&Tag) technique [27] to map the binding sites of *PRDM1α* and *PRDM1β* throughout reprogramming. Given the sequence similarity between *PRDM1α* and *PRDM1β*, commercially available antibodies cannot distinguish them well. We constructed HA-tagged overexpression vectors to overcome this limitation and introduced them into hiF-T cells for naïve reprogramming. However, we observed massive death of cells at the initial and intermediate stages, preventing the acquisition of sufficient iPSC colonies by the end of reprogramming.

**Fig. 5:**
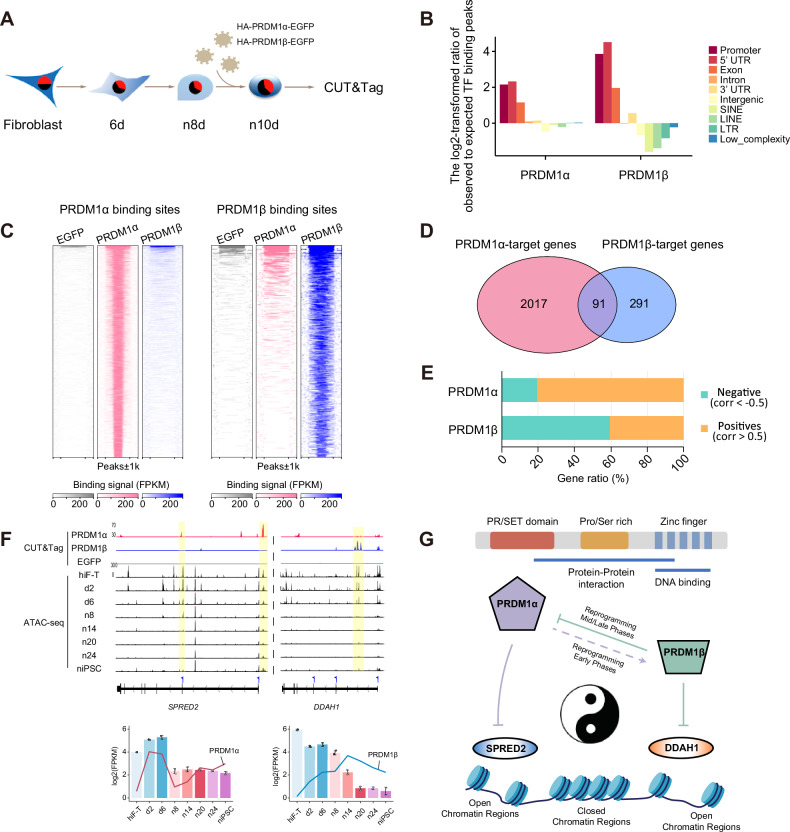
CUT&Tag revealed distinct binding profiles of *PRDM1α* and *PRDM1β.* **A** Schematic experimental design of CUT&Tag analysis during naïve reprogramming. **B** Enrichment of *PRDM1α* and *PRDM1β* binding sites at different genomic features. **C** Heatmap showing the binding signals of *PRDM1α*, *PRDM1β*, and EGFP (empty vector) on *PRDM1α* and *PRDM1β* binding sites. **D** Venn diagram of annotated genes within *PRDM1α* and *PRDM1β* binding sites. **E** Percentage of negative or positive gene expression correlation between *PRDM1α*- and *PRDM1β*-target genes and themselves. **F** The snapshots of the browser view showing chromatin accessibility dynamics and *PRDM1* binding signals near *PRDM1α-* and *PRDM1β-*target genes, *SPRED2* and *DDAH1* (upper panel). The expression levels of *SPRED2* (bar plot) and *PRDM1α* (red line), *DDAH1* (bar plot), and *PRDM1β* (blue line) (bottom panel). **G** Proposed auto-regulation model of the study.

We noted distinct chromatin accessibility profiles and significant transcriptomic changes at day 6 and day 14 of reprogramming, respectively (Fig. 1B and S1C). These observations led us to focus the CUT&Tag analysis between day 6 and day 14 to mitigate potential cellular toxicity while capturing differential binding behaviors. Lentiviral vectors carrying *HA-PRDM1α/PRDM1β-EGFP* were used to infect naïve reprogramming intermediates collected from day 8, with subsequent CUT&Tag analysis conducted after additional reprogramming until day 10 (Fig. 5A). This analysis identified 3,343 binding sites for *PRDM1α* and 471 for *PRDM1β*, with only 53 overlapping sites (Fig. S6A). Detailed examination showed that both *PRDM1α* and *PRDM1β* predominantly occupied promoter and 5’ UTR regions (Fig. 5B), reflecting classical TF binding patterns. *PRDM1β* exhibited more enrichment in promoter and 5’ UTR regions compared to *PRDM1α* (Fig. 5B). The binding signals displayed distinct binding patterns between *PRDM1α* and *PRDM1β* (Fig. 5C and S6B), indicating that *PRDM1β* may function as an auxiliary member to broaden the *PRDM1* regulation network.

We then focused on genes closely located to these binding sites, excluding those with low expression (FPKM < 1), and obtained 2108 genes targeted by *PRDM1α* and 382 by *PRDM1β* (Fig. 5D, Supplementary Table 5). Further analysis showed that 80% of *PRDM1α*-targeted genes positively correlated with *PRDM1α* expression, whereas *PRDM1β*-targeted genes mainly exhibited negative correlation with *PRDM1β* expression (Fig. 5E and S6C). This led us to hypothesize that the functional targets of *PRDM1α* and *PRDM1β* could be identified based on their expression correlation patterns. Based on the previously observed reprogramming phenotypes during the knockdown of *PRDM1α* and *PRDM1β*, we intersected the target genes of *PRDM1α* with the gene set “epithelial-mesenchymal transition” (GO:0001837) and the target genes of *PRDM1β* with the gene set “negative regulation of cell population proliferation” (GO:0008285). This resulted in only one gene, *SPRED2*, for the former, and 6 genes, *ATOH8*, *CITED2*, *DDAH1*, *HGS*, *MAP2K1*, and *SHOC2*, for the latter.

Then we highlighted *SPRED2* and *DDAH1* as downstream targets of *PRDM1α* and *PRDM1β*, respectively (Fig. 5F). SPRED2, a member of the SPRED protein family, is important in the epithelial-mesenchymal transition (EMT) process [28–30]. In mouse ESCs, *Spred2* enhances self-renewal and proliferation [31]. DDAH1 functions as a cysteine hydrolase enzyme that metabolizes endogenous inhibitors of nitric oxide synthase (NOS) [32]. A deficiency in *DDAH1* significantly impairs endothelial cell proliferation [33]. Additionally, *DDAH1* or *SPRED2* knockdown significantly impaired naïve reprogramming accessed by day 20 (Fig. S6D, E). These results suggest that different isoforms of the *PRDM1* gene can target distinct genomic loci, exhibiting a complex and diverse regulatory mechanism.

## Discussion

In this study, we present a detailed roadmap of chromatin accessibility during human somatic reprogramming toward both naïve and primed pluripotency. Our analyses delineate the distinct cell fate trajectories associated with each pluripotency state, offering a valuable framework for exploring the mechanisms underlying reprogramming. We identify *PRDM1* as a crucial transcription factor in chromatin remodeling during reprogramming. Interestingly, *PRDM1* knockdown during human naïve reprogramming results in a unique phenotype characterized by fewer colonies but enhanced morphological quality. Further investigation reveals that the two isoforms of *PRDM1*, *PRDM1α* and *PRDM1β*, exert divergent influences on the reprogramming to naïve pluripotency, with neither isoform being indispensable for the maintenance of naïve pluripotency.

*PRDM1/BLIMP-1*, a transcriptional repressor, plays a crucial role in regulating cell fate decisions during embryonic development and sustaining tissue homeostasis [34, 35], while also functioning as a tumor suppressor [36]. PRDM1α, featuring a full-length PR domain, acts as a tumor suppressor, while PRDM1β, which possesses a truncated PR domain, exhibits oncogenic characteristics [37]. This dichotomy is evidenced by elevated *PRDM1β* expression levels in various cancer cell lines [38–41], suggesting that its oncogenic potential stems from the absence of suppressive activity associated with the full PR domain [38, 40]. Furthermore, *PRDM1β* may directly counteract *PRDM1α* expression, thus facilitating precise control over gene expression. The PRDM protein family, including members such as PRDM2 [42] and PRDM16 [43], showcases a similar yin-yang paradigm in tumor contents observed in PRDM1, characterized by two functionally antagonistic isoforms originating from a single gene. Despite their high sequence similarity, which poses a significant challenge for detailed mechanistic studies, our study provides insights into the role of *PRDM1* in somatic cell reprogramming. This further enriches our understanding of the PRDM protein family’s function, particularly the yin-yang paradigm.

While generating knockdown and overexpression cell lines for *PRDM1α* and *PRDM1β*, we encountered significant challenges in overexpressing *PRDM1β* in naïve hESCs using the same experimental procedures applied for *PRDM1α*. Insights from previous studies on PRDM’s function in cancers suggest that suppression of the full-length PR protein might be a prerequisite for the ectopic expression of a PR-truncated protein [44]. This could account for the difficulties observed in overexpressing *PRDM1β*, although additional experimental evidence is required.

CUT&Tag analysis revealed distinct chromatin-binding profiles for *PRDM1α* and *PRDM1β* during the reprogramming towards human naïve pluripotent state. Recent research based on an engineered *PRDM1-Δexon3* mutant within a human CAR-T cell line illustrates another instance of PR domain truncation within the PRDM1 protein [45]. This mutation resulted in enhanced memory phenotypes, diminished T cell exhaustion, and prolonged effector functionality. Furthermore, applying CUT&RUN to profile chromatin binding across the genome showed similar binding patterns between the mutant and the wildtype *PRDM1*. They were achieved using a PRDM1 antibody recognizing a shared region between the mutant and wild-type forms. This led to the speculation that the observed similarities in binding profiles might be attributed to the presence of other *PRDM1* isoforms in the wild-type samples. To mitigate potential discrepancies arising from the PRDM1 antibody, we employed an HA tag antibody for our CUT&Tag analysis. Further investigation is needed to ascertain whether the observed differences between the studies are due to variations in cellular context or different forms of truncation forms.

Building on the above findings, we propose an auto-regulation model for *PRDM1* during the human naïve reprogramming (Fig. 5G). In this model, *PRDM1α* initially represses *SPRED2* expression. As the expression of *PRDM1α* increases, *PRDM1β* emerges to target both *DDAH1* and *PRDM1α*, thus establishing equilibrium. Knockdown of *PRDM1α* alleviates *SPRED2* inhibition, enhancing pluripotency promotion. Whereas *PRDM1β* knockdown maintains *SPRED2* repression via *PRDM1α*, consequently decreasing pluripotency. Initial knockdown experiments targeting *PRDM1α* and *PRDM1β* exhibited *SPRED2* inhibition relief, promoting pluripotency. Additionally, *PRDM1β* suppresses *DDAH1* expression, resulting in diminished proliferation of reprogramming intermediates with epithelial characteristics. Taken together, the outcome was a phenotype displaying fewer but morphologically superior colonies. Knockdown of *DDAH1* or *SPRED2* did not affect the expression of naïve pluripotency genes at day 10 of reprogramming, but significantly impaired naïve reprogramming by day 20 (Fig. S6D, E). This aligns with our observation that *PRDM1α* and *PRDM1β* function more likely at distinct stages of reprogramming. The similar stage-specific behavior of their downstream targets, *DDAH1* and *SPRED2*, further supports our proposed regulatory model.

### Limitations of study

Although we used the HA-tagged overexpression strategy to delineate the binding profiles of *PRDM1α* and *PRDM1β*, capturing endogenous proteins would provide a more accurate reflection of their functional properties. Developing probes or antibodies that specifically recognize each isoform would greatly enhance this approach.

The current reprogramming process spans over 20 days, during which several critical biological events occur. However, due to constraints in sample size and cost, we are limited to studying only a few time points. Employing single-cell sequencing at multiple time points could offer deeper insights into key signaling pathway interactions during reprogramming, though a more extensive analysis with a larger sample size will be necessary for comprehensive results.

The auto-regulation model we proposed encompasses the entire reprogramming process and is primarily based on phenomena observed at various stages, combined with findings from previous studies. Further experimental validation is required, such as biochemical evidence demonstrating the antagonistic effect of *PRDM1β* on *PRDM1α*.

## Materials and methods

### Cloning

pSicoR (a gift from Tyler Jacks, Addgene plasmid # 11579) was chosen for shRNA expression. The EGFP fragment of pSicoR was replaced by the mCherry fragment. The constitutive expression of shRNA was indicated by the constitutive CMV promoter-driven mCherry fluorescent protein. shRNA specifically targeting specific genes was designed and cloned into the modified pSicoR plasmid for the knockdown experiment (Supplementary Table 6). PRDM1α and PRDM1β coding regions were amplified from human genome DNA, then fused with EGFP and cloned into pCW57.1 (a gift from David Root, Addgene plasmid # 41393) for Dox-inducible overexpression.

### Cell culture

hiF-T cells were cultured in DMEM (Gibco) supplemented with 10% FBS (Gibco), 2 mM L-Glutamine (Millipore), and were passaged by Trypsin-EDTA (0.05%) when the cell confluence reached 90%. Naïve hESCs were cultured in 5iLAF medium containing DMEM/F-12:Neurobasal (1:1) (Gibco), 1× N2 Supplement (Gibco), 1× B27 Supplement (Gibco), 0.5% Knockout Serum Replacement (Gibco), 1× Non Essential Amino Acids (Millipore), 2 mM L-Glutamine (Millipore), 1× Penicillin-Streptomycin (Millipore), 20 ng/mL human LIF (Millipore), 10 ng/mL bFGF (PeproTech), 50 μg/mL bovine serum albumin (BSA, Sigma), 0.1 mM β-mercaptoethanol (Sigma), and the following cytokines and small molecules: 1 μM PD0325901 (Selleck), 1 mM CHIR-99021 (Selleck), 0.5 μM SB590885 (Selleck), 1 μM WH-4-023 (Selleck), 10 μM Y-27632 (Selleck), and 20 ng/mL activin A (PeproTech). Naïve hESCs were cultured on mitomycin C-inactivated MEF feeder cells and were passaged every 4-5 days by Accutase (Gibco). Tests for mycoplasma are performed routinely to ensure negative results.

### Reprogramming

Primed reprogramming was performed in human embryonic stem cell medium (hESM) containing DMEM/F12 (Gibco) with 20% Knockout Serum Replacement (Gibco), 1× Non Essential Amino Acids (Millipore), 2 mM L-Glutamine (Millipore), 1× Penicillin-Streptomycin (Millipore), 8 ng/mL bFGF (PeproTech). The secondary (2°) reprogramming was performed as previously reported [9], hiF-T cells were seeded on feeder cells, then cultured in hESM + Doxycycline (Dox, 1 ng/mL) for 6 days, followed by either culturing as before to generate primed iPSCs or switching to 5iLAF medium to generate naïve iPSCs.

### RT-qPCR

Total RNA was prepared by RNAiso Plus (Takara). cDNA was synthesized by All-In-One 5X RT MasterMix (abm) according to the manufacturer’s instructions. qPCR was performed on the 7500 Fast Real-Time PCR Systems (Applied Biosystems), using TB Green Premix Ex Taq (Takara). The expression level of each gene was normalized to that of HPRT or GAPDH, and all data were measured in triplicate.

### Alkaline phosphatase (AP) staining

AP staining was performed with an Alkaline Phosphatase Assay Kit (Beyotime) according to the manufacturer’s instructions. Briefly, add AP staining solution to fixed cells and incubate at room temperature until the desired staining intensity is reached. Wash stained cells with water or PBS to stop the reaction and monitor staining under a scanner. The experiment was repeated three times. For each one, three images were captured for quantification.

### Immunostaining

Cells were fixed with 4% paraformaldehyde for 20 min at room temperature, then permeabilized by 0.5% Triton X-100/PBS for 15 min. After blocking with 5% BSA/PBS for 30 min at room temperature, cells were incubated with the primary antibody at 4 °C overnight and the appropriate secondary antibody for 60 min at room temperature. Nuclei were stained with DAPI (Beyotime). Images were captured on the confocal microscope (LSM800, Zeiss).

### Western blotting

Cells were lysed in RIPA buffer (KeyGEN BioTECH) on ice for 10 min, then centrifuged for 10 min at 12000 g at 4 °C. The supernatant was mixed with Protein Sample Loading Buffer (Epizyme), heated to 98 °C for 5 min, and then stored at −80 °C. Proteins were separated by SDS-PAGE and transferred onto a PVDF (Millipore) membrane. After blocking with a protein-free rapid blocking buffer (Epizyme), the membrane was incubated with the primary antibody at 4 °C overnight and the corresponding secondary antibody for 60 min at room temperature. The blotting was visualized with a chemiluminescence substrate kit (Thermo Scientific) on the ChemiDoc MP imaging system (Bio-Rad).

### Magnetic-activated cell sorting (MACS)

The pluripotent intermediate population was enriched by MACS with Pluripotent Stem Cell MicroBeads (Miltenyi Biotec) according to the manufacturer’s instructions. Briefly, cells were harvested and resuspended in MACS buffer (DPBS without Ca²+ and Mg²+, with 0.5% BSA and 2 mM EDTA) at a concentration of 20 million cells/mL. Cell suspensions were mixed with CD326 MicroBeads at 20 μL MicroBeads per 2 million cells and incubated in the refrigerator for 15 min. Cells were washed and resuspended with MACS buffer, then separated into CD326+ and CD326− (flow-through) populations using suitable Columns on the MACS Separator.

### Immunoprecipitation (IP) and mass spectrometry (MS)

The previously established overexpression cell lines were used for the IP-MS experiment. The cells were harvested after 24 h Dox treatment. Immunoprecipitation was performed using ANTI-FLAG M2 Affinity Gel (Sigma) according to the manufacturer’s instructions. The elution was analyzed by SDS-PAGE electrophoresis. The excision gel was submitted to PTM Biolabs for further mass spectrometry.

### Bulk mRNA-seq

Total RNA was extracted by RNAiso Plus (Takara). Bulk mRNA-seq libraries were prepared by KAPA mRNA HyperPrep Kit (Kapa Biosystems) following the manufacturer’s instructions. Libraries were submitted to paired-end sequencing on the NovaSeq 6000 platform (Illumina) according to the manufacturer’s instructions at Berry Genomics Corporation. In the preparation of sequencing libraries, we aim to use three biological replicates wherever possible. However, if the final concentration of any biological replicate is found to be too low, we opt for two reliable replicates for sequencing to err on the side of caution.

### ATAC-seq

ATAC-seq was performed as previously reported [8] by TruePrep DNA Library Prep Kit V2 for Illumina (Vazyme). Briefly, a total of 50,000-10,000 cells were resuspended in 200 μL lysis buffer (10 mM Tris–HCl pH 7.4, 10 mM NaCl, 3 mM MgCl2, 0.15% NP-40) and placed on the ice for 10-12 min lysis. The suspension was centrifuged for 5 min at 500 g at 4 °C, and then 40 μL transposition reaction mix (8 μL 5 × TTBL, 10 μL TTEmix V5, and 22 μL nuclease-free H2O) was added to the pellet. Samples were mixed well and incubated at 37 °C for 30 min. After adding 10 μL 5 × TS to stop the reaction, ATAC-seq libraries were PCR amplified upon the appropriate cycles and submitted to gel electrophoresis. Libraries were purified with NucleoSpin Gel and PCR Clean-Up (MN). Library concentration was determined by Qubit dsDNA HS Kit (Invitrogen). The resultant ATAC-seq libraries, each consisting of two biological replicates, were performed paired-end sequencing on the NovaSeq 6000 platform (Illumina) according to the manufacturer’s instructions at Berry Genomics Corporation.

### Lentivirus production

293T cells were cultured in DMEM (Gibco) supplemented with 10% FBS (Gibco) and 2 mM L-Glutamine (Millipore). The verified lentiviral vectors (pSicoR or pCW57.1 based) were co-transfected with the packaging plasmids pMD2.G (a gift from Didier Trono, Addgene plasmid #12260) and psPAX2 (a gift from Didier Trono, Addgene plasmid # 12260) into 293 T cells using VigoFect (Vigorous Biotech) according to the manufacturer’s instructions. After 10–12 h, the transfection reagents were removed and replaced with fresh medium. The virus-containing supernatant was harvested 48 h post medium changing, filtered with 0.45 μm sterile filters (Millipore), and concentrated by 10% (m/v) PEG8000 (Sigma) rotation at 4 °C overnight. The lentiviral particles were collected by centrifugation for 30 min at 4000 g at 4 °C.

### Protein structure modeling by Alphafold2

The structure of PRDM1β truncated PR domain was predicted by Alphafold2 using MMseqs2. The crystal structure of PRDM1α (PRDM1) was obtained in Protein Database Bank (PDB): 3DAL. Pymol 2.5.0 was used to align and visualize the PR domain (PRDM1α) and truncated PR domain (PRDM1β).

### CUT&Tag

CUT&Tag was performed as previously described [27] by Hyperactive Universal CUT&Tag Assay Kit for Illumina (Vazyme). Briefly, a total of 50,000 cells were harvested, washed by Wash Buffer, and centrifuged for 5 min at 300 × g at room temperature. 10 μL pre-activated ConA beads were added per sample and incubated at room temperature for 10 min. The supernatant was discarded, and the cell-beads mixture was resuspended with 50 μL Antibody Buffer with 1 μg primary antibody (Anti-HA, Cell Signaling Technology #3724S) and incubated at 4 °C overnight. Then the primary antibody was removed, added 50 μL Dig-wash Buffer with 0.5 μg secondary antibody (Anti-Rb IgG, antibodies-online #ABIN101961), and rotated at room temperature for 1 h. After washing 3 times with 200 μL Dig-wash buffer, 2 μL PA/G–Tnp was added with 98 μL Dig-300 buffer and rotated at room temperature for 1 h, then washed 3 times with 200 μL Dig-wash buffer. 40 μL Dig-300 Buffer was mixed with 10 μL 5 × TTBL, then added and incubated at 37 °C for 1 h. The fragmented DNA was extracted with DNA Extract Beads and amplified upon the appropriate cycles. Libraries were purified with 2 × AMPure XP (Beckman Coulter). Library concentration was determined by Qubit dsDNA HS Kit (Invitrogen). The resultant CUT&Tag libraries were performed paired-end sequencing on the NovaSeq 6000 platform (Illumina) according to the manufacturer’s instructions at Nanjing Jiangbei New Area.

### Statistics and reproducibility

No data were excluded from the analyses. Data collection and analysis were not performed blind to the conditions of the experiments, but data analyses were performed with identical parameters and software. Data representation and statistical analyses were performed using R software (v4.0.0), GraphPad Prism 9, and Excel. All error bars indicate the standard error of the mean (± SEM). Results were analyzed by unpaired two‐tailed Student’s t‐test, as indicated in figure legends. All qPCR experiments were performed with three technical replicates.

### ATAC-seq data analysis

Sequencing reads were first trimmed to remove adapters and then mapped to the human genome (UCSC hg19) using Bowtie2 (v2.3.4.3) with the parameters: -N 0 -X 2000 -q -t --no-mixed --no-discordant --no-unal [46]. Mitochondrial DNA and low-confidence reads were filtered out using SAMtools (v1.7). Duplicated reads were removed using sambamba (v0.6.8) [47]. Genome-wide signals were calculated using a 25-bp window and normalized to the uniquely mapped fragments using ‘bamCoverage’ from deepTools (v2.5.7) [48]. The programs ‘computeMatrix’ and ‘multiBigwigSummary’ of deepTools (v2.5.7) were used to compute the ATAC-seq signals over the region of interest. Genomic features annotation and prediction of transcription factor motifs were performed using Homer (4.11.1) [49].

Peaks were identified by macs2 (v2.1.1) [50], and peaks from different samples were combined into one set of genomic coordinates using BEDTools [51] with the parameter -d 100. Subsequently, this set was employed to generate random background signals as pseudo-input. Regions exhibiting log2-transformed ATAC-seq signals above 4.2 are considered to be true open chromatin regions, with a 0.01 false positive rate compared to the pseudo-input. All downstream analysis is based on this threshold value of 4.2; if the ATAC-seq is below this value, it is annotated as ‘closed’ and above ‘open’. Specifically, regions that remained open throughout the reprogramming process were defined as permanently open (PO). Regions that transitioned from closed in fibroblasts to open as reprogramming proceeded were defined as closed to open (CO). Conversely, regions that transitioned from open to closed were defined as open to closed (OC).

Transiently open or closed regions were analyzed with unsupervised clustering using the R package Mfuzz. The nearest transcription start site (TSS) located within 10 kb of these ATAC-seq peaks were identified and subjected to downstream analysis, including gene expression quantification and gene ontology (GO) analysis.

### RNA-seq data analysis

Sequencing reads were first trimmed to remove adapters and then mapped to the annotated human transcripts (UCSC hg19) using Hisat2 (v2.1.0) with the parameters: --dta --no-mixed --no-discordant --no-unal [52]. Mapped reads with high confidence were kept for further analysis using SAMtools (v1.7) with the parameters: -bf 0 × 2 -q 20 [53]. The retained reads were subsequently assembled into transcripts guided by the UCSC gtf annotation files using Stringtie (v1.3.4) [54]. The expression level of each gene was quantified as FPKM (fragments per kilobase per million mapped reads). Differential expression genes were defined with fold-change > 2 and *q* value < 0.05 using R package Ballgown. To define the upregulated genes during naïve reprogramming, the fold change in gene expression compared to hiF-T should be greater than 5, with a p-value less than 0.0001 at each stage of reprogramming, and the gene’s FPKM should be greater than 5 at that time point.

### CUT&Tag data analysis

The process from data quality control (QC) to peak calling is consistent with ATAC-seq data analysis. The ‘computeMatrix’, ‘plotHeatmap’ and ‘plotProfile’ programs of deepTools (v2.5.7) were used to compute the protein binding signals within the regions of interest.

### Gene ontology analysis

The R package ClusterProfiler (v 4.2.2) was used to identify Gene Ontology (GO) terms using databases Biological Functions [55].

## Supplementary information

Supplementary Data

Supplymentary Table 1

Supplymentary Table 2

Supplymentary Table 3

Supplymentary Table 4

Supplymentary Table 5

Supplymentary Table 6

Original Blots

## Data Availability

The ATAC-seq datasets generated in this study are available at GEO: GSE255859. The bulk RNA-seq datasets generated in this study are available at GEO: GSE255861. The CUT&Tag datasets generated in this study are available at GEO: GSE255860. The accession numbers for the RNA-seq data of published cell lines are E-MTAB-2856, GSE59435, GSE166401, CNP0001454, GSE150772, and GSE174771.
