# Supplementary material for: Chromatin landscape dynamics during reprogramming towards human naïve and primed pluripotency reveals the divergent function of PRDM1 isoforms
Source: Cell Death Discov. 2024 Nov 19;10:474. doi: 10.1038/s41420-024-02230-w (PMC11576854; doi:10.1038/s41420-024-02230-w)
Supplement: Supplementary file 1 — Supplementary Data [file 41420_2024_2230_MOESM1_ESM.pdf]

Supplementary Data

Chromatin landscape dynamics during reprogramming towards human naïve and primed pluripotency reveals the divergent function of *PRDM1* isoforms

Jianfeng Zhou[1,2,6], Mingyue Guo[2,3,6], Guang Yang[2,4], Xinyu Cui[2,4], Jindian Hu[1,2], Tan Lin[1,2], Hong Wang[1,2], Shaorong Gao[1,2*], Cizhong Jiang[2,4*], Liping Wang[2,5*], Yixuan Wang[1,2*]

* Correspondence: Yixuan Wang, Liping Wang, Cizhong Jiang, Shaorong Gao,

Email: wangyixuan@tongji.edu.cn
wlp@tongji.edu.cn
czjiang@tongji.edu.cn
gaoshaorong@tongji.edu.cn

Fig S1

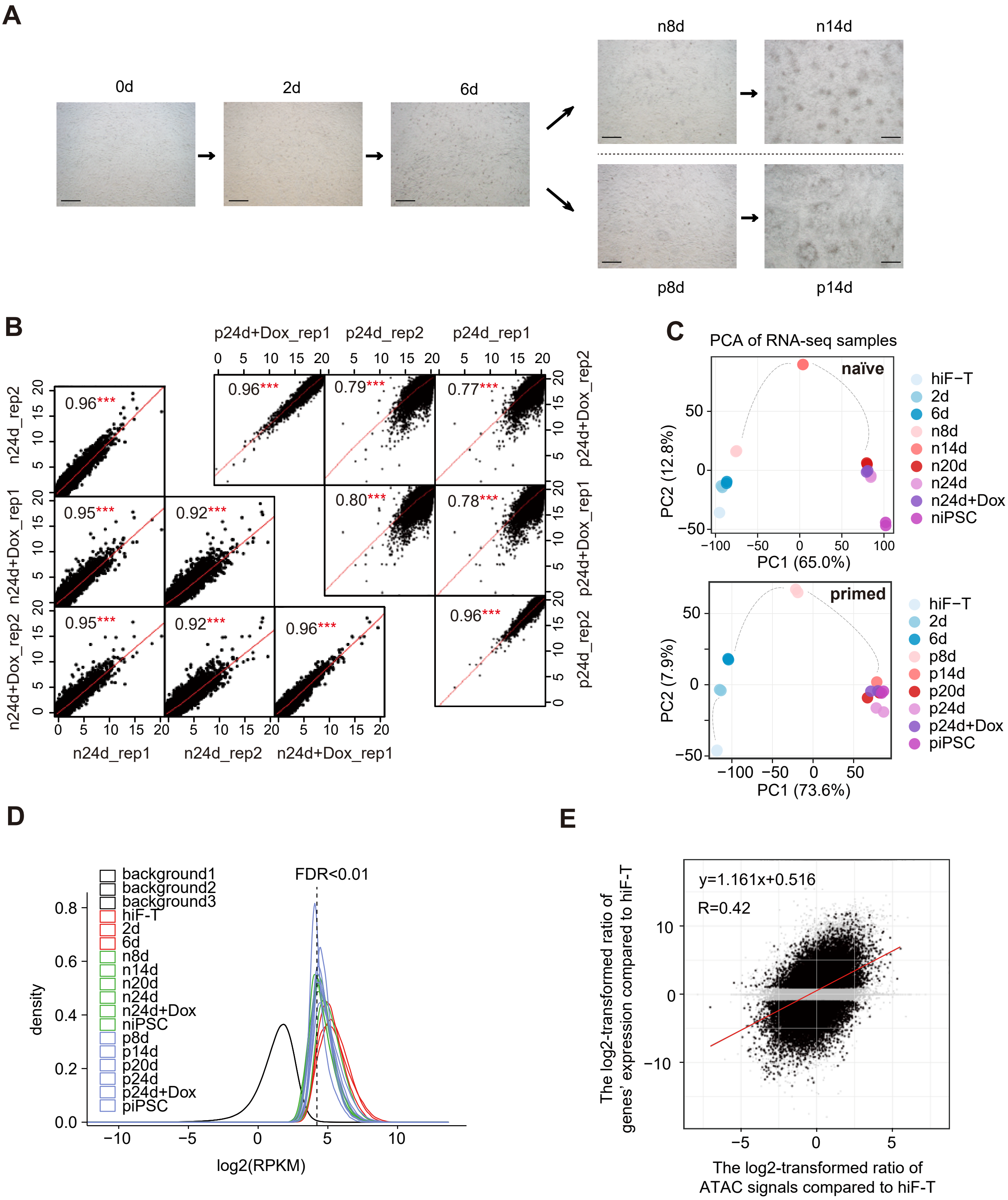
A
0d
2d
6d
n8d
n14d
p8d
p14d
B
p24d+Dox_rep1
p24d_rep2
p24d_rep1
n24d_rep2
n24d+Dox_rep1
n24d+Dox_rep2
p24d+Dox_rep2
p24d+Dox_rep1
p24d_rep2
n24d_rep1
n24d_rep2
n24d+Dox_rep1
0.96***
0.95***
0.92***
0.95***
0.92***
0.96***
0.96***
0.79***
0.77***
0.80***
0.78***
0.96***
C
PCA of RNA-seq samples
naïve
PC2 (12.8%)
PC1 (65.0%)
hiF−T
2d
6d
n8d
n14d
n20d
n24d
n24d+Dox
niPSC
primed
PC2 (7.9%)
PC1 (73.6%)
hiF−T
2d
6d
p8d
p14d
p20d
p24d
p24d+Dox
piPSC
D
FDR<0.01
density
log2(RPKM)
background1
background2
background3
hiF-T
2d
6d
n8d
n14d
n20d
n24d
n24d+Dox
niPSC
p8d
p14d
p20d
p24d
p24d+Dox
piPSC
E
y=1.161x+0.516
R=0.42
The log2-transformed ratio of genes' expression compared to hiF-T
The log2-transformed ratio of ATAC signals compared to hiF-T

Fig. S1 (Related to Fig. 1) Reprogramming process snapshots and data quality control.

(A) Cellular morphology changes during human 2° hiF-T reprogramming. Scale bar, 100μm.

(B) Scatter plot showing the correlation between 24d biological replicates and samples with or without continued Dox supplement until day 24 during reprogramming. Each point in the figure represents the FPKM value for an ATAC-seq signal peak. Pearson's correlation coefficient and p-value are indicated. *** p-value < 0.001.

(C) PCA on the RNA-seq data collected from cells at different stages of naïve and primed reprogramming.

(D) Distribution of ATAC-seq signals in cells at different stages of reprogramming versus random background signals. A threshold of 4.2 was chosen to give a false positive rate of 0.01.

(E) Scatter plot showing the correlation between genes' expression and ATAC-seq signal at the gene promoters. The fitted linear equation and Pearson's correlation coefficient are shown.

Fig S2

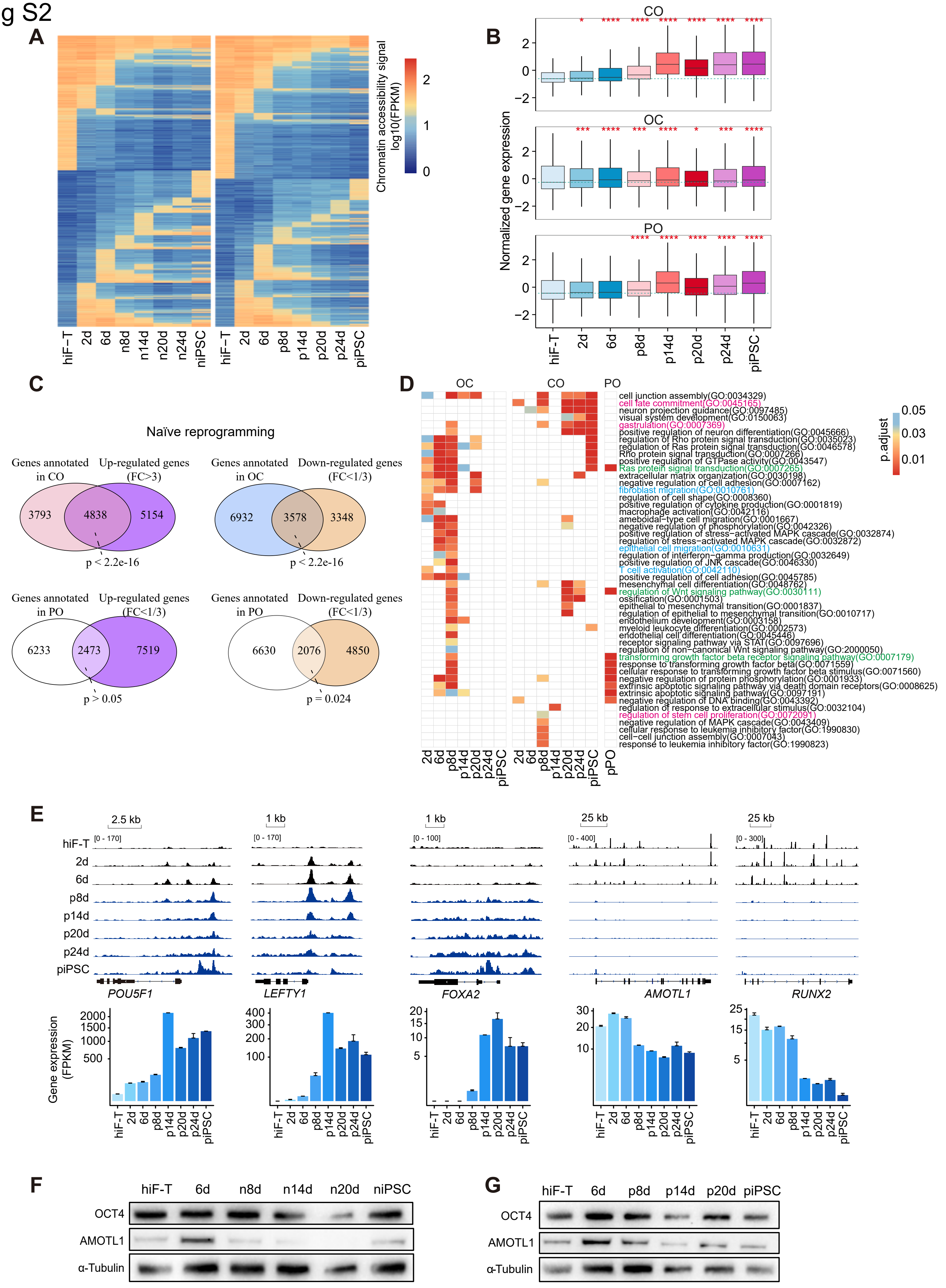
A
hiF−T
2d
6d
n8d
n14d
n20d
n24d
niPSC
p8d
p14d
p20d
p24d
piPSC
Chromatin accessibility signal log10(FPKM)
2
1
0
B
CO
OC
PO
Normalized gene expression
C
Naïve reprogramming
Genes annotated in CO
Up-regulated genes (FC>3)
3793
4838
5154
p < 2.2e-16
Genes annotated in OC
Down-regulated genes (FC<1/3)
6932
3578
3348
p < 2.2e-16
Genes annotated in PO
Up-regulated genes (FC<1/3)
6233
2473
7519
p > 0.05
Genes annotated in PO
Down-regulated genes (FC<1/3)
6630
2076
4850
p = 0.024
D
OC
CO
PO
pPO
p.adjust
0.05
0.03
0.01
cell junction assembly(GO:0034329)
cell fate commitment(GO:0045165)
neuron projection guidance(GO:0097485)
visual system development(GO:0150063)
gastrulation(GO:0007369)
positive regulation of neuron differentiation(GO:0045666)
regulation of Rho protein signal transduction(GO:0035023)
regulation of Ras protein signal transduction(GO:0046578)
Rho protein signal transduction(GO:0007266)
positive regulation of GTPase activity(GO:0043547)
Ras protein signal transduction(GO:0007265)
extracellular matrix organization(GO:0030198)
negative regulation of cell adhesion(GO:0007162)
fibroblast migration(GO:0010761)
regulation of cell shape(GO:0008360)
positive regulation of cytokine production(GO:0001819)
macrophage activation(GO:0042116)
ameboidal−type cell migration(GO:0001667)
negative regulation of phosphorylation(GO:0042326)
positive regulation of stress−activated MAPK cascade(GO:0032874)
regulation of stress−activated MAPK cascade(GO:0032872)
epithelial cell migration(GO:0010631)
regulation of interferon−gamma production(GO:0032649)
positive regulation of JNK cascade(GO:0046330)
T cell activation(GO:0042110)
positive regulation of cell adhesion(GO:0045785)
mesenchymal cell differentiation(GO:0048762)
regulation of Wnt signaling pathway(GO:0030111)
ossification(GO:0001503)
epithelial to mesenchymal transition(GO:0001837)
regulation of epithelial to mesenchymal transition(GO:0010717)
endothelium development(GO:0003158)
myeloid leukocyte differentiation(GO:0002573)
endothelial cell differentiation(GO:0045446)
receptor signaling pathway via STAT(GO:0097696)
regulation of non−canonical Wnt signaling pathway(GO:2000050)
transforming growth factor beta receptor signaling pathway(GO:0007179)
response to transforming growth factor beta(GO:0071559)
cellular response to transforming growth factor beta stimulus(GO:0071560)
negative regulation of protein phosphorylation(GO:0001933)
extrinsic apoptotic signaling pathway via death domain receptors(GO:0008625)
extrinsic apoptotic signaling pathway(GO:0097191)
negative regulation of DNA binding(GO:0043392)
regulation of response to extracellular stimulus(GO:0032104)
regulation of stem cell proliferation(GO:0072091)
negative regulation of MAPK cascade(GO:0043409)
cellular response to leukemia inhibitory factor(GO:1990830)
cell−cell junction assembly(GO:0007043)
response to leukemia inhibitory factor(GO:1990823)
E
2.5 kb
1 kb
25 kb
[0 - 170]
[0 - 100]
[0 - 400]
[0 - 300]
POU5F1
LEFTY1
FOXA2
AMOTL1
RUNX2
Gene expression (FPKM)
F
hiF-T
6d
n8d
n14d
n20d
niPSC
OCT4
AMOTL1
α-Tubulin
G
p8d
p14d
p20d
piPSC

Fig. S2 (Related to Fig. 1) ATAC-seq and mRNA-seq revealed highly dynamic chromatin changes during human naïve and primed reprogramming.

(A) Chromatin regions with transiently open and closed peaks in the reprogramming time course (uncategorized). Log10-transformed FPKM values are used to represent the degree of chromatin accessibility at a given region.

(B) Box plots showing the normalized expression levels of genes within 10 kb of PO, CO, and OC regions in primed reprogramming. The green dashed line indicates the median gene expression level corresponding to the hiF-T stage. Mann-Whitney U test versus the levels in hiF, *, $p < 0.05$; **, $p < 0.01$; ***, $p < 0.001$; ****, $p < 0.0001$.

(C) Venn diagram showing the overlap of differential expression genes in the three defined regions in naïve of Fig 1C. Genes annotated to regions were defined as those with the nearest transcription start site (TSS) within 10 kb of each region. Differentially expressed genes were identified as those exhibiting a 3-fold up-regulation or down-regulation at any time point during the reprogramming process. Statistical significance was assessed using the hypergeometric test.

(D) GO analysis on all genes within 10 kb of the PO, CO, and OC regions in primed reprogramming.

(E) Representative gene expression and chromatin accessibility in primed reprogramming; pluripotency genes, *POU5F1*; developmental patterning genes, *LEFTY1*, *FOXA2*; somatic genes, *AMOTL1*, *RUNX2*.

(F) Western blot verification of OCT4, AMOTL1 in naïve reprogramming. α-Tubulin was set as the control.

(G) Western blot verification of OCT4, AMOTL1 in primed reprogramming. α-Tubulin was set as the control.

Fig S3

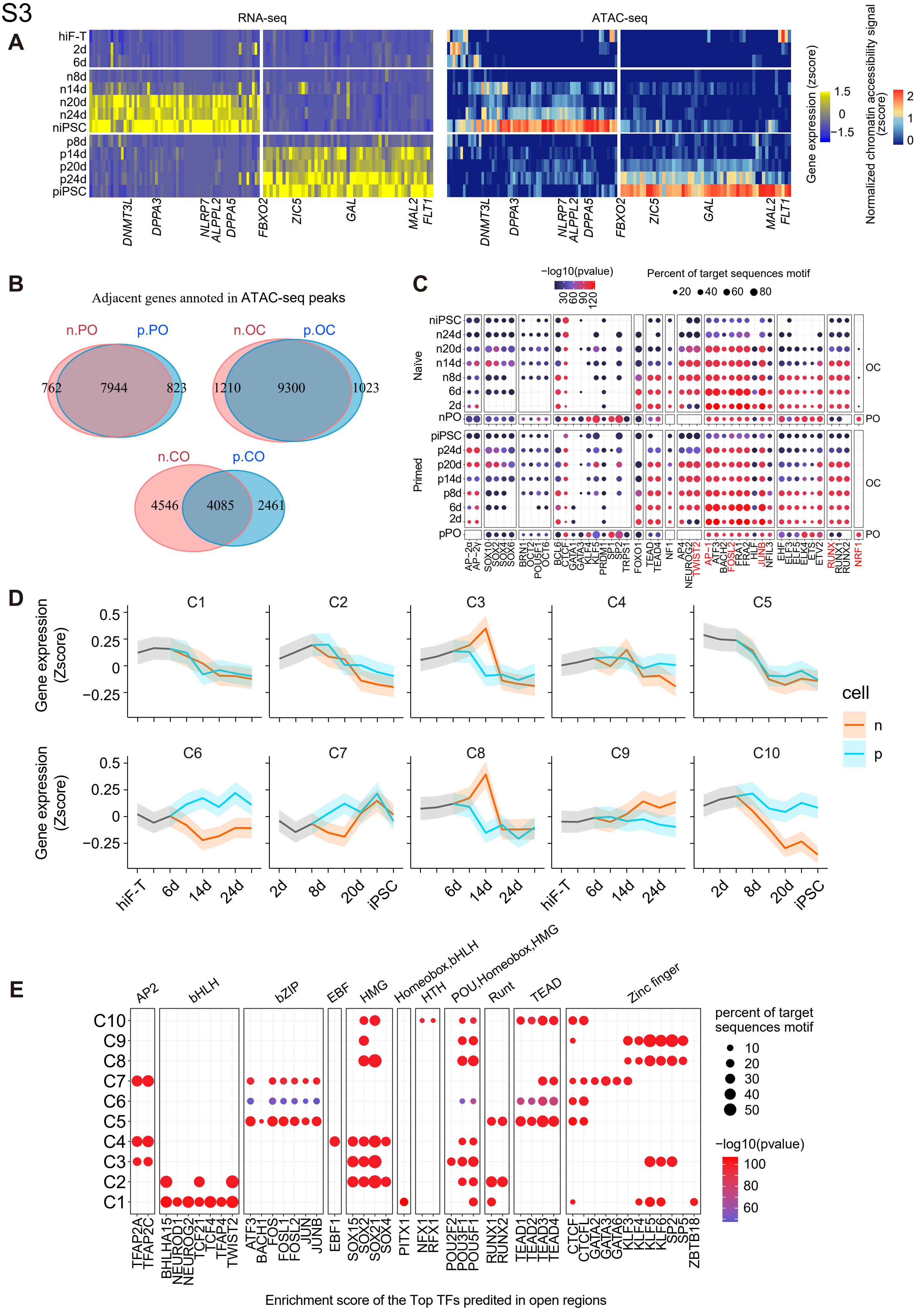
A
RNA-seq
ATAC-seq
hiF-T
2d
6d
n8d
n14d
n20d
n24d
niPSC
p8d
p14d
p20d
p24d
piPSC
DNMT3L
DPPA3
NLRP7
ALPPL2
DPPA5
FBXO2
ZIC5
GAL
MAL2
FLT1
Gene expression (zscore)
1.5
0
−1.5
Normalized chromatin accessibility signal (zscore)
2
1
0
B
Adjacent genes annoted in ATAC-seq peaks
n.PO
p.PO
762
7944
823
n.OC
p.OC
1210
9300
1023
n.CO
p.CO
4546
4085
2461
C
−log10(pvalue)
30
60
90
120
Percent of target sequences motif
20
40
60
80
Naive
Primed
nPO
pPO
OC
PO
AP−2d
AP−2Y
SOX10
SOX4
SOX6
BRN1
OCT4
POU5F1
OCT6
BCL6
CTCF
GATA1
GATA3
KLF4
KLF5
PRDM1
SP1
SP2
TRPS1
FOXO1
TEAD
TEAD4
NF1
AP4
NEUROG2
TWIST2
AP−1
ATF3
BACH2
FOSL2
FRA1
FRA2
HLF
JUNB
NFIL3
EHF
ELF3
ELF5
ELK4
ETS
ETV2
RUNX
RUNX1
RUNX2
NRF1
D
C1
C2
C3
C4
C5
C6
C7
C8
C9
C10
Gene expression (Zscore)
0.5
0.25
0
−0.25
cell
n
p
hiF-T
6d
14d
24d
2d
8d
20d
iPSC
E
AP2
bHLH
bZIP
EBF
HMG
Homeobox,bHLH
HTH
POU,Homeobox,HMG
Runt
TEAD
Zinc finger
TFAP2A
TFAP2C
BHLHA15
NEUROD1
NEUROG2
NE
TCF21
TCF4
TFAP4
TWIST2
ATF3
BACH1
FOS
FOSL1
FOSL2
JUN
JUNB
EBF1
SOX15
SOX2
SOX21
SOX4
PITX1
NFX1
RFX1
POU2F2
POU3F1
POU5F1
RUNX1
RUNX2
TEAD1
TEAD2
TEAD3
TEAD4
CTCF
CTCFL
GATA2
GATA3
GATA6
KLF3
KLF4
KLF5
KLF6
SP2
SP5
ZBTB18
percent of target sequences motif
10
20
30
40
50
−log10(pvalue)
100
80
60
Enrichment score of the Top TFs predited in open regions

Fig. S3 (Related to Fig. 2) TF motif analysis of identified genomic region clusters.

(A) The chromatin accessibility levels of regions specifically opened or closed during naïve and primed reprogramming (left panel), along with the expression levels of adjacent genes (right panel).

(B) Venn diagram showing the overlap of adjacent genes in PO, OC, and CO regions between naïve and primed reprogramming.

(C) Transcription factor motifs significantly enriched at OC and PO regions in naïve and primed reprogramming.

(D) Gene expression dynamics in the 10 clusters of transiently open or closed chromatin regions given in Fig 2G. Solid lines and ribbons represent the mean of standardized gene expression levels across clusters ± s.d.

(E) The bubble chart illustrating the most significant enrichment of transcription factor motifs in each cluster as depicted in Fig 2F.

Fig S4

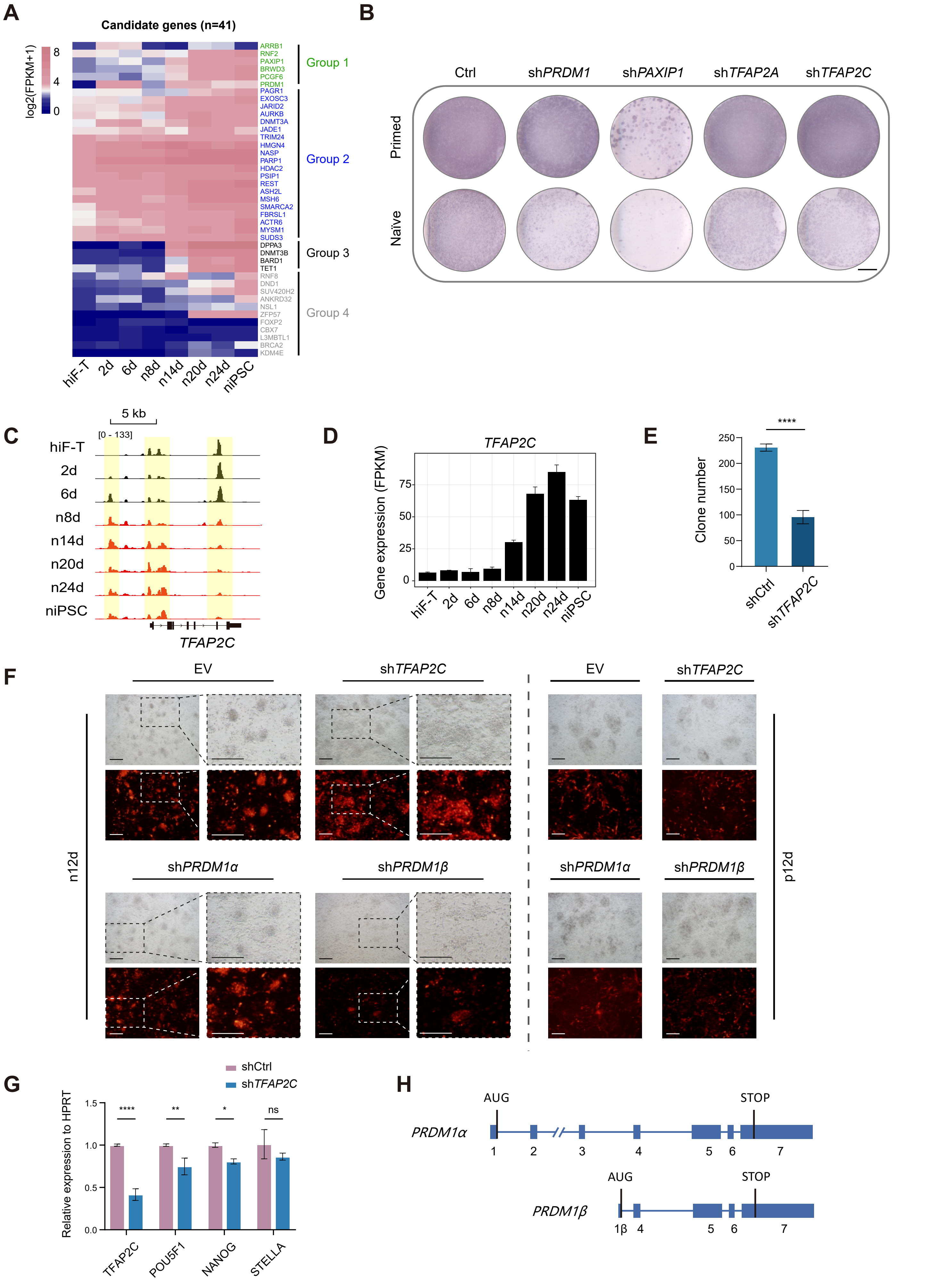
A
Candidate genes (n=41)
log2(FPKM+1)
8
4
0
ARRB1
RNF2
PAXIP1
BRWD3
PCGF6
PRDM1
PAGR1
EXOSC3
JARID2
AURKB
DNMT3A
JADE1
TRIM24
HMGN4
NASP
PARP1
HDAC2
PSIP1
REST
ASH2L
MSH6
SMARCA2
FBRSL1
ACTR6
MYSM1
SUDS3
DPPA3
DNMT3B
BARD1
TET1
RNF8
DND1
SUV420H2
ANKRD32
NSL1
ZFP57
FOXP2
CBX7
L3MBTL1
BRCA2
KDM4E
Group 1
Group 2
Group 3
Group 4
hiF-T
2d
6d
n8d
n14d
n20d
n24d
niPSC
B
Ctrl
shPRDM1
shPAXIP1
shTFAP2A
shTFAP2C
Primed
Naïve
C
5 kb
[0 - 133]
hiF-T
2d
6d
n8d
n14d
n20d
n24d
niPSC
TFAP2C
D
TFAP2C
Gene expression (FPKM)
75
50
25
0
hiF-T
2d
6d
n8d
n14d
n20d
n24d
niPSC
E
****
Clone number
250
200
150
100
50
0
shCtrl
shTFAP2C
F
EV
shTFAP2C
EV
shTFAP2C
n12d
shPRDM1α
shPRDM1β
shPRDM1α
shPRDM1β
p12d
G
shCtrl
shTFAP2C
Relative expression to HPRT
1.5
1.0
0.5
0.0
****
**
*
ns
TFAP2C
POU5F1
NANOG
STELLA
H
AUG
STOP
PRDM1α
1
2
3
4
5
6
7
AUG
STOP
PRDM1β
1β
4
5
6
7

Fig. S4 (Related to Fig. 3) *PRDM1* and *TFAP2C* were important for naïve reprogramming.

(A) Heatmap showing the expression levels of 41 candidate genes (identified in Fig. 3A) in naïve reprogramming. These genes were classified into four groups according to expression dynamics.

(B) Alkaline Phosphatase (AP) staining of selected candidate genes' knockdown experiments. The purple color indicates the presence of stem cells. The experiment was repeated three times. Scale bar, 8mm.

(C) The snapshots of the browser view showing chromatin accessibility dynamics near *TFAP2C* in naïve reprogramming.

(D) Gene expression levels of *TFAP2C* in naïve reprogramming.

(E) Clone numbers under sh*TFAP2C* in naïve reprogramming. The experiment was repeated three times; **** adjusted. p-value < 0.0001.

(F) Bright field and fluorescent images of naïve reprogramming intermediates in n12d upon sh*TFAP2C*, sh*PRDM1α* and sh*PRDM1β,* empty vector was set as Ctrl (left panel). Bright field and fluorescent images of primed reprogramming intermediates in p12d upon sh*TFAP2C*, sh*PRDM1α* and sh*PRDM1β* (right panel). Scale bar, 100µm.

(G) Relative expression of *TFAP2C*, *POU5F1*, *NANOG* and *STELLA* upon sh*TFAP2C* treatment via qPCR in naïve reprogramming. n = 3; Two-way ANOVA, *, adjusted p-value = 0.0303; ** adjusted p-value = 0.0043; ****, adjusted p-value < 0.0001.

(H) Gene structures of *PRDM1α* and *PRDM1β*.

Fig S5

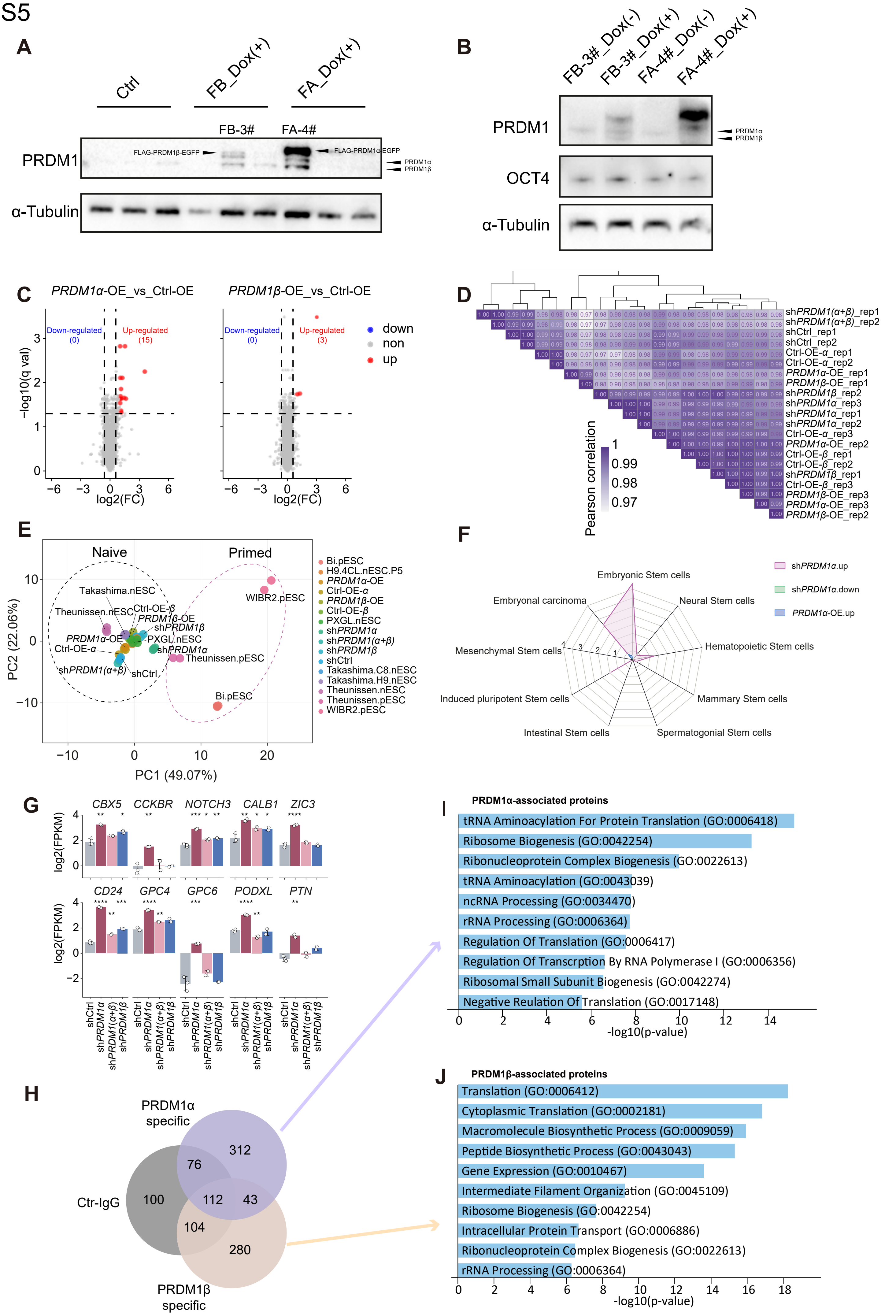

A
Ctrl
FB_Dox(+)
FA_Dox(+)
FB-3#
FA-4#
PRDM1
FLAG-PRDM1β-EGFP
FLAG-PRDM1α-EGFP
PRDM1α
PRDM1β
α-Tubulin
B
FB-3#_Dox(-)
FB-3#_Dox(+)
FA-4#_Dox(-)
FA-4#_Dox(+)
PRDM1
OCT4
α-Tubulin
C
PRDM1α-OE_vs_Ctrl-OE
PRDM1β-OE_vs_Ctrl-OE
Down-regulated (0)
Up-regulated (15)
Up-regulated (3)
-log10(q val)
log2(FC)
down
non
up
D
Pearson correlation
E
Naive
Primed
PC1 (49.07%)
PC2 (22.06%)
Bi.pESC
H9.4CL.nESC.P5
PRDM1α-OE
Ctrl-OE-α
PRDM1β-OE
Ctrl-OE-β
PXGL.nESC
shPRDM1α
shPRDM1(α+β)
shPRDM1β
shCtrl
Takashima.C8.nESC
Takashima.H9.nESC
Theunissen.nESC
Theunissen.pESC
WIBR2.pESC
F
Embryonic Stem cells
Neural Stem cells
Hematopoietic Stem cells
Mammary Stem cells
Spermatogonial Stem cells
Intestinal Stem cells
Induced pluripotent Stem cells
Mesenchymal Stem cells
Embryonal carcinoma
shPRDM1α.up
shPRDM1α.down
PRDM1α-OE.up
G
CBX5
CCKBR
NOTCH3
CALB1
ZIC3
CD24
GPC4
GPC6
PODXL
PTN
log2(FPKM)
shCtrl
shPRDM1α
shPRDM1(α+β)
shPRDM1β
H
PRDM1α specific
Ctr-IgG
PRDM1β specific
312
76
100
112
43
104
280
I
PRDM1α-associated proteins
tRNA Aminoacylation For Protein Translation (GO:0006418)
Ribosome Biogenesis (GO:0042254)
Ribonucleoprotein Complex Biogenesis (GO:0022613)
tRNA Aminoacylation (GO:0043039)
ncRNA Processing (GO:0034470)
rRNA Processing (GO:0006364)
Regulation Of Translation (GO:0006417)
Regulation Of Transcrption By RNA Polymerase I (GO:0006356)
Ribosomal Small Subunit Biogenesis (GO:0042274)
Negative Reulation Of Translation (GO:0017148)
-log10(p-value)
J
PRDM1β-associated proteins
Translation (GO:0006412)
Cytoplasmic Translation (GO:0002181)
Macromolecule Biosynthetic Process (GO:0009059)
Peptide Biosynthetic Process (GO:0043043)
Gene Expression (GO:0010467)
Intermediate Filament Organization (GO:0045109)
Ribosome Biogenesis (GO:0042254)
Intracellular Protein Transport (GO:0006886)
Ribonucleoprotein Complex Biogenesis (GO:0022613)
rRNA Processing (GO:0006364)
-log10(p-value)

Fig. S5 (Related to Fig. 4) Perturbation of *PRDM1α* and *PRDM1β* showed negligible effects on naïve pluripotency maintaining.

(A) Western blot analysis of PRDM1 overexpression and pluripotent OCT4 in naïve hESCs. Three different cell lines in each group were detected. α-Tubulin was set as the control. FA and FB represented Dox inducible overexpression cell lines of *PRDM1α* and *PRDM1β* respectively. EGFP was used in the overexpression vector as a reporter gene. FA-4# and FB-3# were chosen for later experiments.

(B) Western blot verification of OE *PRDM1α*, OE *PRDM1β* in naïve hESCs. α-Tubulin was set as the control.

(C) Volcano plot of differential expression genes between OE *PRDM1α*, OE *PRDM1β* versus Ctrl in naïve hESCs.

(D) Pearson correlation of the RNA-seq data collected from naïve hESCs with overexpression or knockdown of *PRDM1* isoforms. Biological replicates >= 2.

(E) PCA on transcriptome data from naïve hESCs with overexpression or knockdown of *PRDM1* isoforms and published naïve hESCs and primed hESCs.

(F) StemChecker results of up- and down-regulation gene set upon sh*PRDM1α*, and up-regulation gene sets when OE *PRDM1α*-in naïve hESCs. The gene count in those sets is more than 10.

(G) The expression levels of genes associated with embryonic stem cells are given in (F). T-test versus the levels in shCtrl, * $p < 0.05$, ** $p < 0.01$, *** $p < 0.001$, **** $p < 0.0001$ and empty means non-significant.

(H) Venn diagram of PRDM1α or PRDM1β-specific proteomes in naïve hESCs. IgG was used as a background group.

(I) GO analysis of PRDM1α-specific proteome.

(J) GO analysis of PRDM1β-specific proteome.

Fig S6

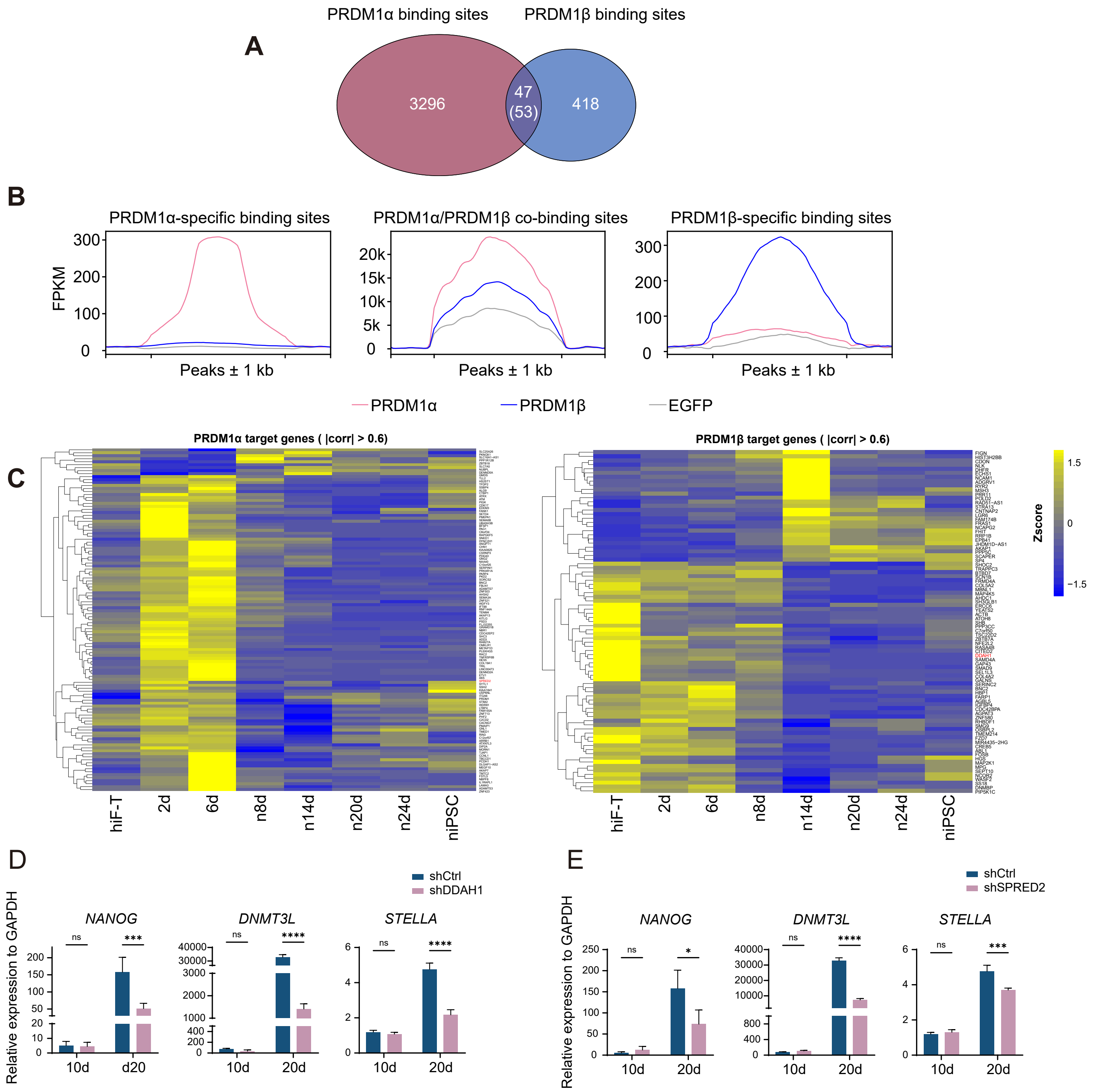
A
PRDM1α binding sites
PRDM1β binding sites
3296
47
(53)
418
B
PRDM1α-specific binding sites
PRDM1α/PRDM1β co-binding sites
PRDM1β-specific binding sites
FPKM
Peaks ± 1 kb
PRDM1α
PRDM1β
EGFP
C
PRDM1α target genes (|corr| > 0.6)
PRDM1β target genes (|corr| > 0.6)
Zscore
hiF-T
2d
6d
n8d
n14d
n20d
n24d
niPSC
D
shCtrl
shDDAH1
Relative expression to GAPDH
NANOG
DNMT3L
STELLA
ns
***
****
10d
d20
20d
E
shCtrl
shSPRED2
Relative expression to GAPDH
NANOG
DNMT3L
STELLA
ns
*
****
***
10d
20d

Fig. S6 (Related to Fig. 5) *PRDM1α* and *PRDM1β* target different gene loci during naïve reprogramming.

(A) Venn diagram of *PRDM1α* and *PRDM1β* binding sites.

(B) Binding profiles of *PRDM1α*, *PRDM1β*, and EGFP (empty vector) signals on *PRDM1* binding sites.

(C) Heatmap showing the expression levels of genes showing high correlation with *PRDM1α* or *PRDM1β.*

(D) Relative expression of *NANOG, DNMT3L* and *STELLA* upon sh*DDAH1* treatment via qPCR in naïve reprogramming. n = 3; Two-way ANOVA, ***, adjusted p-value < 0.001; ****, adjusted p-value < 0.0001.

(E) Relative expression of *NANOG, DNMT3L* and *STELLA* upon sh*SPRED2* treatment via qPCR in naïve reprogramming. n = 3; Two-way ANOVA, *, adjusted p-value < 0.05; ***, adjusted p-value = 0.001; ****, adjusted p-value < 0.0001.

Supplementary Table 1: Summary of data used in the article.

Supplementary Table 2: The identified CO and C8 peaks list, related to Figure 3.

Supplementary Table 3: The up-regulated genes during naïve reprogramming, related to Figure 3.

Supplementary Table 4: The interacting proteins with PRDM1α and PRDM1β, related to Figure 5.

Supplementary Table 5: *PRDM1α* and *PRDM1β* targeting genes list, related to Figure 5.

Supplementary Table 6: shRNA sequences used in this study.

Supplementary Original Blots
