# Supplementary figures and images for: Chromatin landscape dynamics during reprogramming towards human naïve and primed pluripotency reveals the divergent function of PRDM1 isoforms
Source: Cell Death Discov. 2024 Nov 19;10:474. doi: 10.1038/s41420-024-02230-w (PMC11576854; doi:10.1038/s41420-024-02230-w)

Fig. S2F

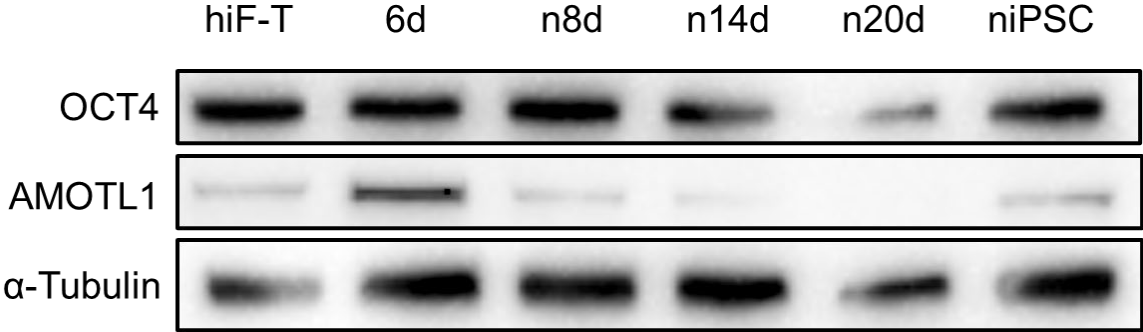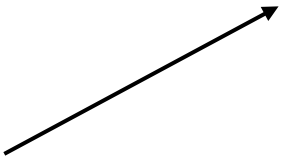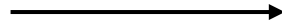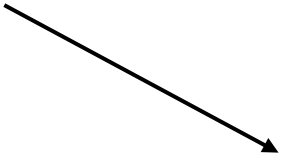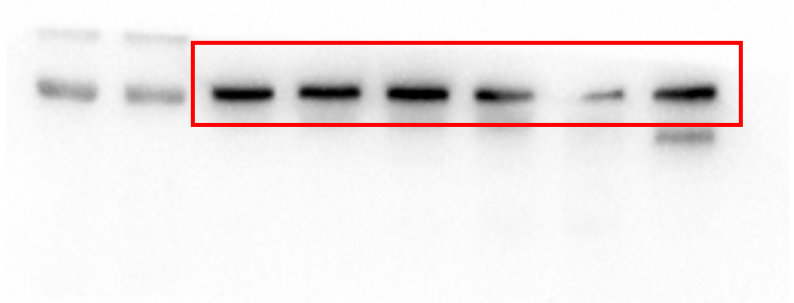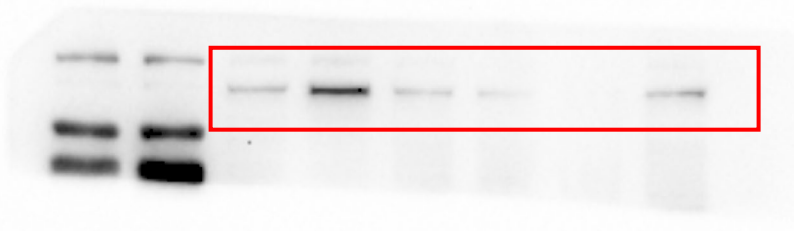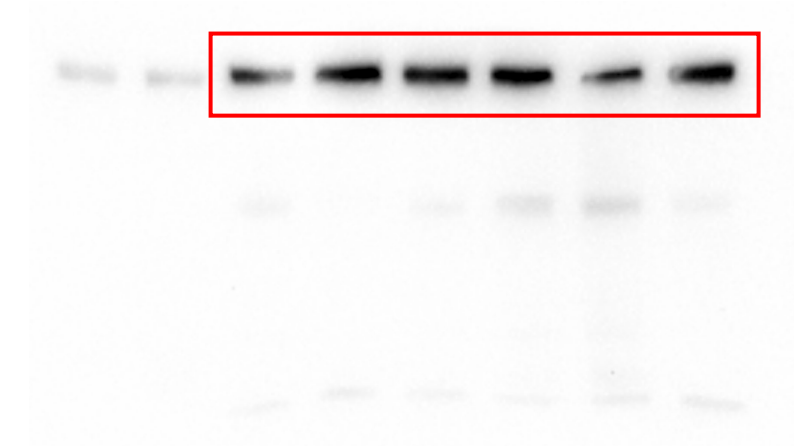

Fig. S2G

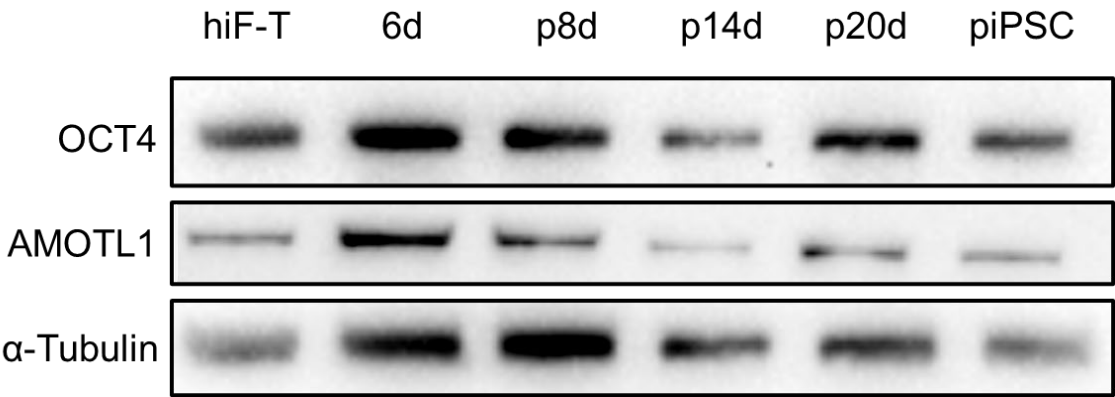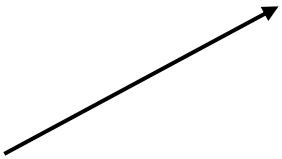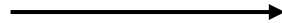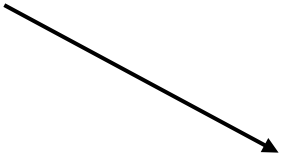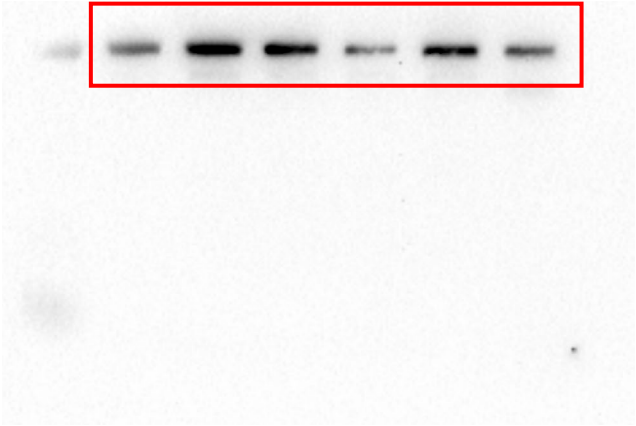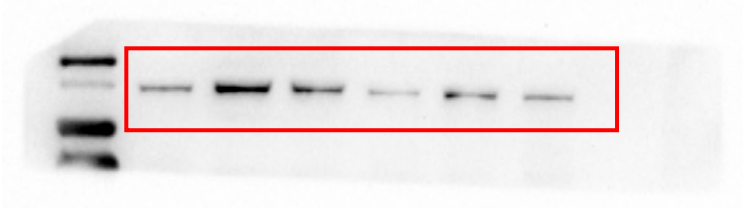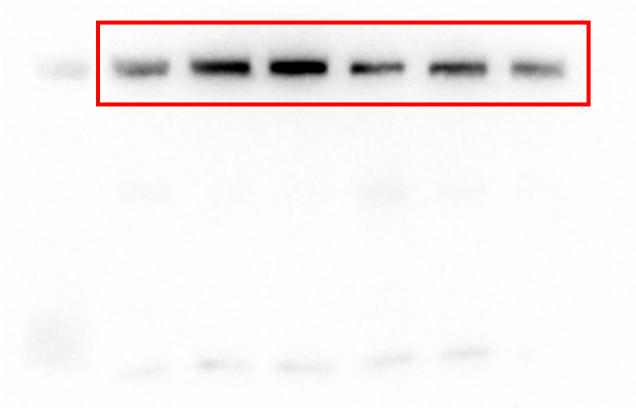

Uncropped western blots

Fig. S5A

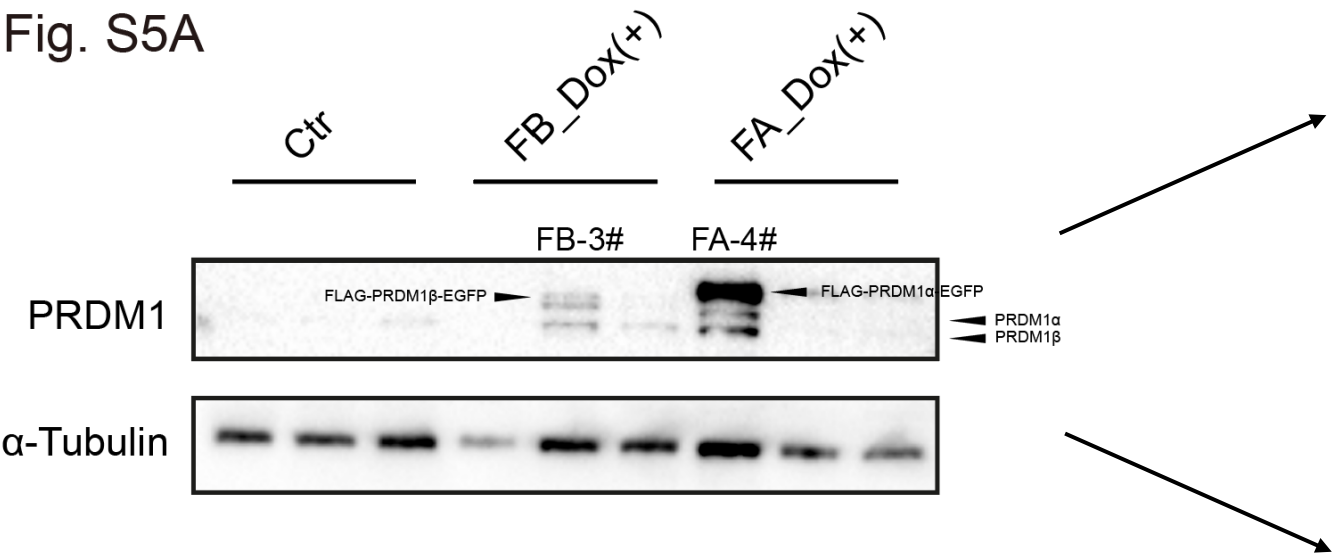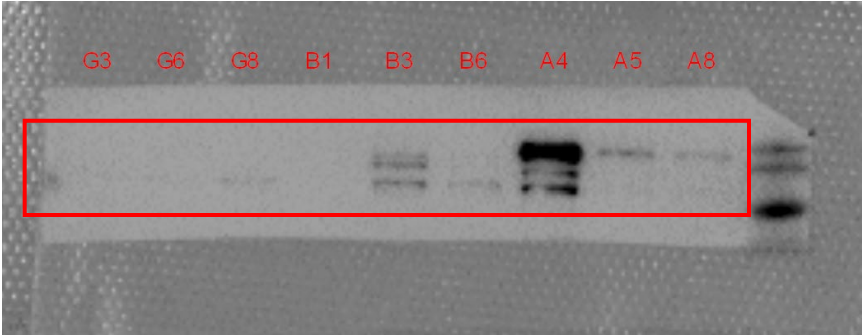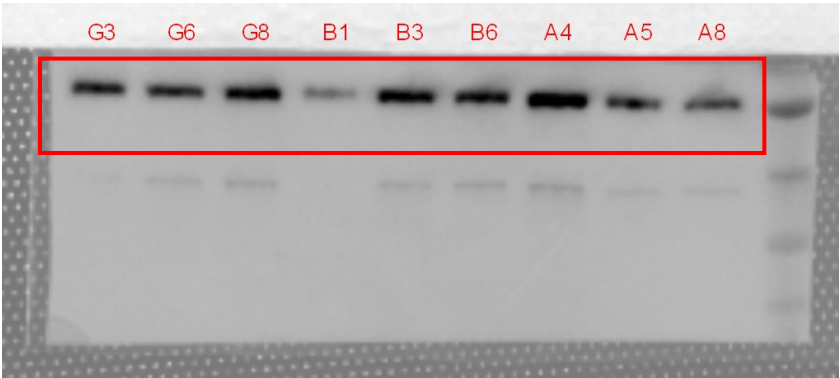

Uncropped western blots

Fig. S5B

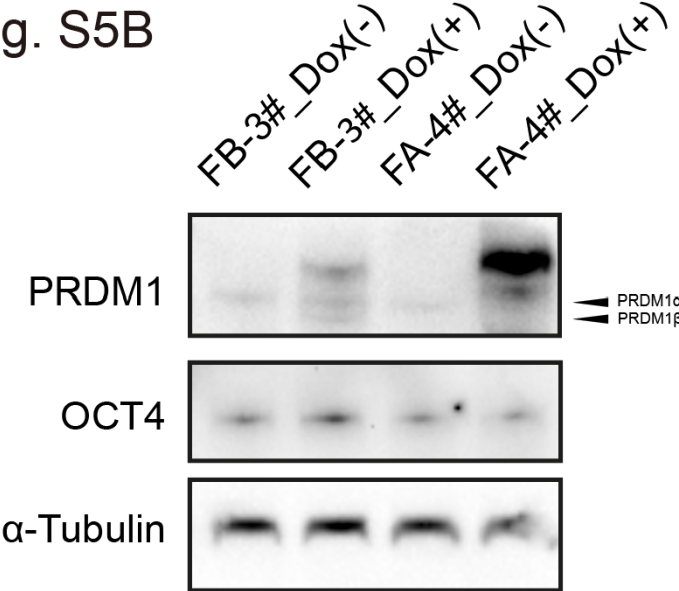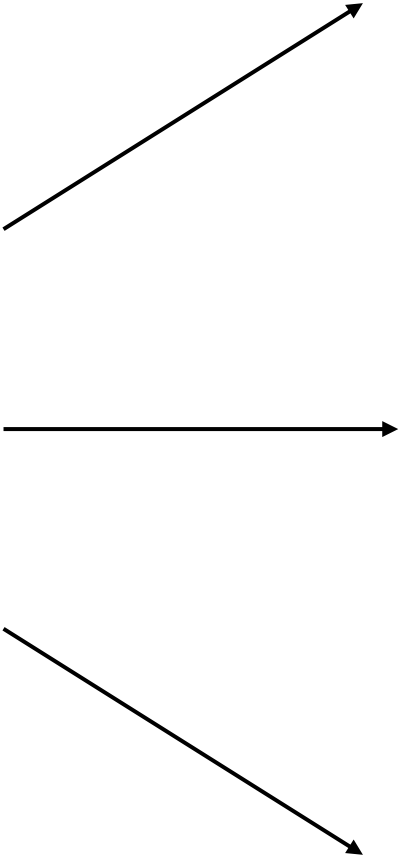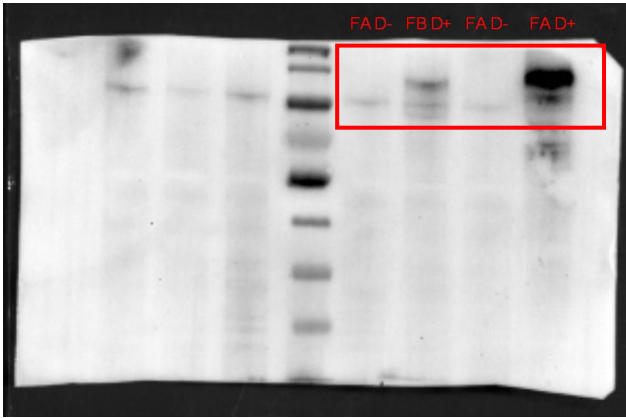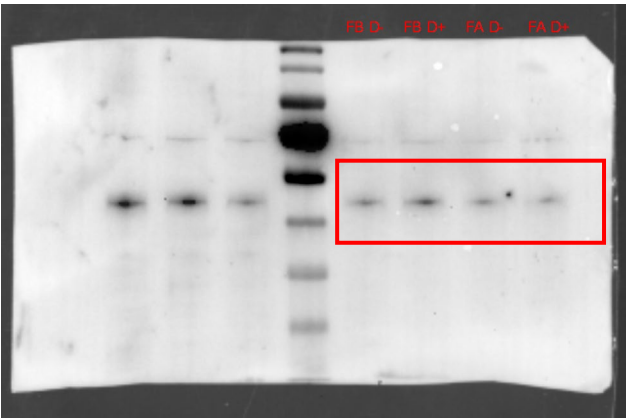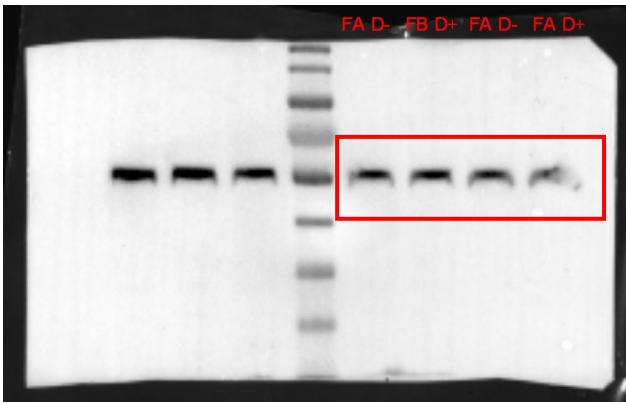

Supplement: Supplementary file 8 — Original Blots [file 41420_2024_2230_MOESM8_ESM.pdf]
